# Designer Liposomic Nanocarriers Are Effective Biofilm Eradicators

**DOI:** 10.1021/acsnano.2c04232

**Published:** 2022-08-26

**Authors:** Monika Kluzek, Yaara Oppenheimer-Shaanan, Tali Dadosh, Mattia I. Morandi, Ori Avinoam, Calanit Raanan, Moshe Goldsmith, Ronit Goldberg, Jacob Klein

**Affiliations:** † Department of Materials and Interfaces, Weizmann Institute of Science, Rehovot 76100, Israel; ‡ Department of Chemical Research Support, Weizmann Institute of Science, Rehovot 76100, Israel; § Department of Biomolecular Sciences, Weizmann Institute of Science, Rehovot 76100, Israel; ∥ Department of Veterinary Resources, Weizmann Institute of Science, Rehovot 76100, Israel

**Keywords:** liposome functionalization, zwitterionic polymer−bacteria membrane interactions, drug delivery, antibiotic resistance, biofilm

## Abstract

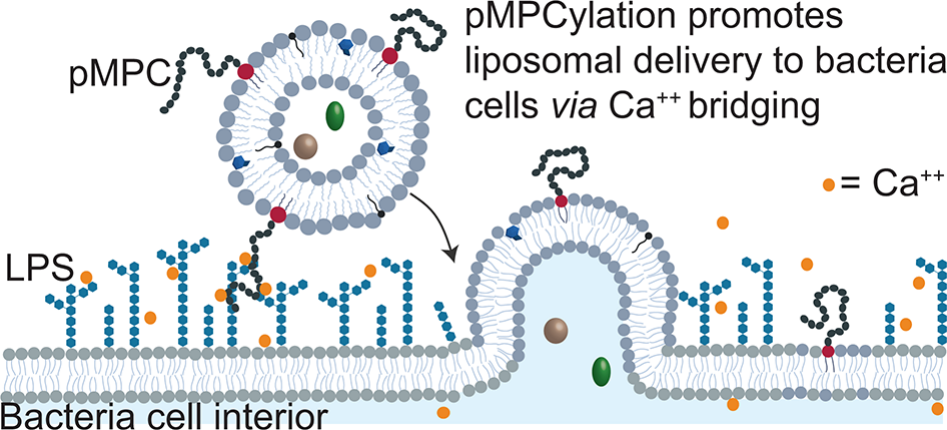

Drug delivery via nanovehicles is successfully employed in several clinical settings, yet bacterial infections, forming microbial communities in the form of biofilms, present a strong challenge to therapeutic treatment due to resistance to conventional antimicrobial therapies. Liposomes can provide a versatile drug-vector strategy for biofilm treatment, but are limited by the need to balance colloidal stability with biofilm penetration. We have discovered a liposomic functionalization strategy, using membrane-embedded moieties of poly[2-(methacryloyloxy)ethyl phosphorylcholine], pMPC, that overcomes this limitation. Such pMPCylation results in liposomic stability equivalent to current functionalization strategies (mostly PEGylation, the present gold-standard), but with strikingly improved cellular uptake and cargo conveyance. Fluorimetry, cryo-electron, and fluorescence microscopies reveal a far-enhanced antibiotic delivery to model *Pseudomonas aeruginosa* biofilms by pMPC-liposomes, followed by faster cytosolic cargo release, resulting in significantly greater biofilm eradication than either PEGylation or free drug. Moreover, this combination of techniques uncovers the molecular mechanism underlying the enhanced interaction with bacteria, indicating it arises from bridging by divalent ions of the zwitterionic groups on the pMPC moieties to the negatively charged lipopolysaccharide chains emanating from the bacterial membranes. Our results point to pMPCylation as a transformative strategy for liposomal functionalization, leading to next-generation delivery systems for biofilm treatment.

Nanovehicles, prominently including liposomes, are effectively used for drug delivery in several contexts,^1^ but bacterial infections forming surface-adherent microbial communities, or biofilms, common in living tissues and on synthetic surfaces, are notoriously resistant to delivery of antimicrobial agents. This is due to their highly developed and adaptive defense and communication mechanisms,^2,3^ which act partly via physical barriers limiting their penetration, as well as limited cell entry due to low membrane permeability.^3,4^

A crucial requirement,^5^ therefore, to enhance biofilm eradication is the development of new nanodelivery systems that can effectively penetrate biofilms’ structure and, by improved interaction with unique bacteria membrane characteristics, release their drug cargo directly into the biofilm-embedded cells. To date, the leading drug vectors of choice fulfilling these requirements are lipid vesicles, or liposomes,^6^ due to their low immunogenicity, firm safety profiles,^7^ and the ability to encapsulate both lipophilic and hydrophilic compounds.^8−10^ The usage of liposomes has significantly improved the therapeutic index for a range of biomedical applications by stabilizing active agents, overcoming obstacles to cellular and tissue uptake and improving biodistribution of compounds to target sites in vivo, all while maintaining high safety.^10^ Despite many advantages of liposomal formulations as nanocarriers, liposomes have an intrinsically low colloidal stability (which results in short shelf life) and low penetration through the biofilm matrix, which strongly limit their efficacy in eradicating biofilms.^11^ The most common strategy utilized to improve these drawbacks is to introduce surface functionalization via conjugation of hydrophilic polymers (polyethylene glycol (PEG) being the current gold standard) to the surface of the carriers to obtain sterically stabilized liposomes.^12,13^ However, such PEGylation has been shown to drastically reduce interaction with the target cells^14−16^ and therefore significantly limits their applicability in successful biofilm eradication.^17^

We have now designed a liposomal nanocarrier able to overcome the limitations of both nonfunctionalized and current surface-functionalized liposomal carriers in delivery to biofilms. In this, moieties consisting of lipid-conjugated phosphocholinated polymers, (poly[2-(methacryloyloxy)ethyl phosphorylcholine], pMPC)^18,19^ are inserted into the liposomal membranes, and in addition, we optimized our liposomes for biofilm treatment with respect both to their size and charge. The phosphocholine-like structure of the MPC monomers is zwitterionic, and an important hypothesis of this study is that their resulting highly dipolar nature would result in stronger interactions with the heterogeneously charged bacterial cells than would be the case for stabilizing moieties such as PEG which are only weakly polar. At the same time, such pMPC moieties are highly hydrated^20^ and have been shown to suppress nonspecific protein adsorption,^21,22^ while pMPC-coating of either chitosan nanoparticles or DNase resulted in their markedly higher diffusion within the biofilms matrix.^23,24^ Here, we exploit the ability of our pMPC functionalization to rapidly penetrate biofilms, together with the delivery-efficacy of pMPCylated liposomes arising from their specific interaction with the bacterial cell surface, to effectively eradicate *Pseudomonas aeruginosa* (PA) biofilms, a widely used model for bacterial infections. Using a combination of fluorimetry and fluorescence and cryo-electron (cryo-TEM) microscopies, we reveal the MPC–membrane interaction mechanism enabling the higher delivery and eradication efficiency of our carriers.

Our results show unambiguously that pMPCylated liposomes are not only colloidally stable and biocompatible but also possess a high affinity to bacteria cells and, by bypassing the membrane barrier, release their cargo directly into the cytosol in both laboratory and clinical *P. aeruginosa* strains. We attribute this enhanced cell-penetration ability to a divalent-cation-mediated bridging of the MPC moieties with polysaccharides on the bacterial membrane, leading to the liposomes’ adhesion to the cell surface and subsequent fusion releasing the cargo into the cytosol. These properties of pMPCylated carriers to successfully deliver cargo result in significantly higher biofilm eradication levels than when using equivalent free drugs or other functionalized (PEGylated) liposomes. In summary, our liposomal functionalization strategy overcomes the limitations of current liposomal carriers for biofilm treatment, providing both penetration abilities through the physical barriers erected by bacterial communities together with efficient delivery of antimicrobial agents directly into the bacterial cells.

## Results and Discussion

### Calcium Strongly Mediates Adhesion between pMPC Moieties and the Bacterial Membrane

As noted, a central hypothesis is that the dipolar nature of the MPC monomers enables a stronger affinity with the bacterial cells. To examine this, we must take account of any multivalent ions whose presence could modulate the interactions between the zwitterionic MPC and negative charges known to be present on bacteria surfaces. Indeed, it is known that calcium and other divalent ions are highly abundant at the outer lipopolysaccharide (LPS)-exposing membrane surfaces of Gram-negative bacteria, where they play a crucial structural role,^25−28^ while phosphocholine groups are known to possess a high calcium-binding affinity.^29^ To elucidate this interplay, we examined directly how Ca^2+^ affects pMPCylated, large unilamellar vesicles (LUVs), and comparable PEGylated vesicles.

**Figure 1:**
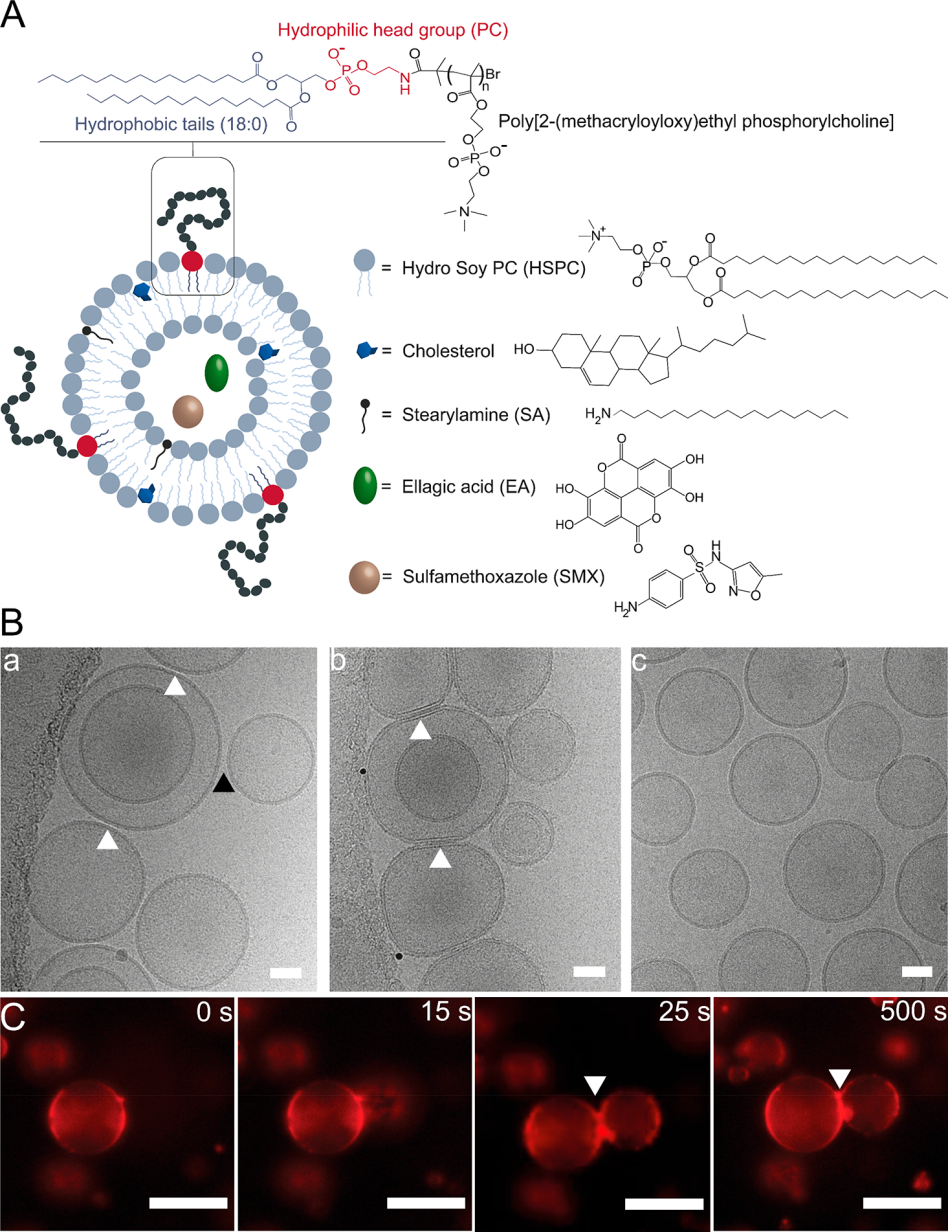
(A) Schematic illustration of the pMPC-functionalized liposomes, depicting pMPC polymer conjugated to phosphatidylethanolamine (DSPE) lipid present at the liposome surface at 5% (mol/mol).^18^ The pMPCylated liposomes are composed of HSPC with 40% (mol/mol) cholesterol and 5% (mol/mol) SA. Liposomes were loaded with antimicrobial agents: either SMX on their own or together with EA. (B) Representative cryo-TEM images of unloaded pMPCylated LUVs after 1 h incubation in (a) 10 mM and (b) 40 mM Ca(Ac)_2_ solution and (c) PEG-LUVs in 40 mM solution of Ca(Ac)_2_. White arrows indicate adhesion points, black arrow indicates no adhesion. Scale bar, 50 nm. (C) Confocal microscopy images of time-lapse acquisition of pMPC-GUVs stained with DiI dye (red), interacting upon addition of 40 mM Ca(Ac)_2_. Scale bar, 20 μm.

LUVs composed of saturated hydrogenated soybean phosphatidylcholine (HSPC) and containing 5% (mol/mol) pMPC-conjugated distearylphosphorylethanolamine (DSPE) (*M*_pMPC_ = 5 kDa), Figure 1A, were prepared as previously described.^18^ To improve mechanical flexibility and cargo retention, liposomes were doped with 40% cholesterol,^30−32^ and 5% (mol/mol) stearylamine (SA) was added to the final composition to offset the negative charge associated with the DSPE (Figure 1A) and for increased drug loading stability (Supporting Information (SI), Table S1).^33−35^ Likewise, identical LUVs but incorporating PEG-conjugated DSPE (*M*_PEG_ = 5 kDa) instead of the pMPC moieties were similarly prepared for comparison.

The pMPCylated LUVs have long-term stability against aggregation, similar to PEGylated vesicles, showing uniform size distribution peaking at ca. 180 nm diameter and with low, constant polydispersity for at least 15 weeks (PDI < 0.1, Figure S1). Unfunctionalized liposomes, which would provide a direct comparison of the pMPC or PEG affinity toward bacteria membranes, showed poor colloidal stability and rapid aggregation (Figure S6).

In investigating the carriers’ affinity and interaction, we thus focused on comparing PEGylation and pMPCylation, as both would be suitable for delivery applications. Moreover, pMPC functionalization and the incorporation of the SA into the vesicles did not compromise their biocompatibility (Figure S2).

Cryo-TEM was used to image pMPCylated and PEGylated LUVs following 1 h incubation with an increasing concentration of Ca(Ac)_2_ (Figure 1B). At both 10 mM and 40 mM Ca(Ac)_2_ concentrations, pMPC-LUVs display adhesion, with 60% and 98% incidence (Figure S3), respectively. The interaction between vesicles changes from weak adhesion at 10 mM (Figure 1B(a), arrows) to a highly flattened contact region at 40 mM, indicating strong adhesion (Figure 1B(b), white arrows). In contrast, PEG-LUVs incubated at 40 mM Ca(Ac)_2_ do not adhere at all and have stochastically distributed separations between adjacent vesicles (Figure 1B(c) and Figure S3). These results demonstrate the role of Ca^2+^ in bridging pMPC moieties (but not PEG) on neighboring vesicles. A detailed examination reveals that the gaps between adhering outer membranes are ca. 4 nm (for both Ca^2+^ concentrations, Figure S3), consistent with two interdigitating opposing layers of 5 kDa pMPC-chains bridged by calcium.^36^ The adhesion process is rapid, as visualized by live imaging microscopy on pMPC-functionalized giant vesicles (GUVs) (Figure 1C). At 40 mM Ca(Ac)_2_, GUVs display long-lasting adhesion occurring within 25 s of contact of the opposing membranes (Figure 1C), whereas in the absence of divalent ions conditions, GUVs do not stably adhere to each other, even upon stochastic contact (Figure S4).

**Figure 2:**
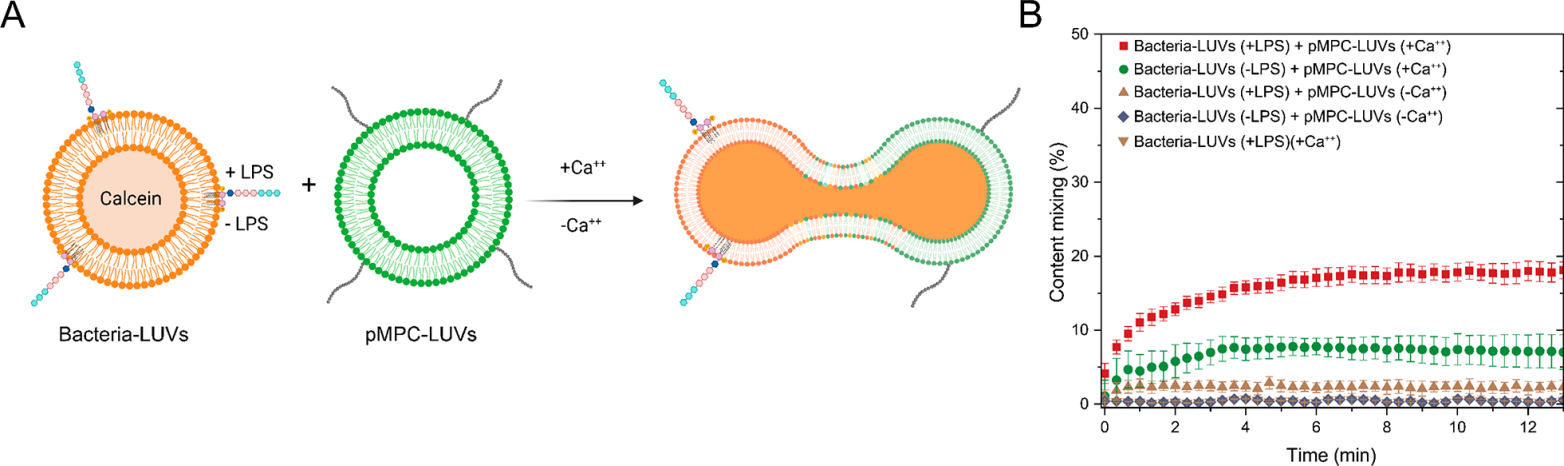
pMPC-LUVs interacting with bacteria-mimicking membranes in differing calcium ions and LPS conditions. (A) Graphical illustration of the bacteria-LUVs fusion with pMPC-liposomes measured with calcein dequenching assay. (B) Profile of calcein content mixing assay between bacteria-liposomes (w or w/o LPS) and calcein-free pMPC-liposomes in the presence and absence of calcium ions (final concentration: 0.72 mM). Results are an average of three independent experiments.

To further corroborate the role of calcium cations in pMPC interaction with bacterial membranes, we investigated calcein release of LUVs composed of bacteria phospholipids extract (either with membrane-incorporated LPS or LPS-free) incubated with pMPC-LUVs under differing calcium concentration (Figure 2A).

Our results show that bacteria-mimicking liposomes containing LPS, when mixed with pMPC-LUVs, display a significant increase in calcein fluorescence in the presence of a low calcium concentration (0.72 mM), reaching 18% content mixing within 5 h (Figure 2B). Contrariwise, incubation in calcium-free conditions causes only a negligible increase in calcein fluorescence of bacterial liposomes (∼2.3%) upon incubation with pMPC-LUVs. Similarly, LPS-free bacteria-mimicking liposomes show moderate content mixing (∼7%) upon interaction with pMPC-liposomes under calcium conditions, while in calcium-free milieu only minimal calcein release occurs (Figure 2B). Overall, these results indicate pMPC liposomes would display enhanced interaction with the LPS- and Ca^2+^-containing bacterial membranes.

### pMPCylated LUVs Attach Strongly to Bacterial Membranes

**Figure 3:**
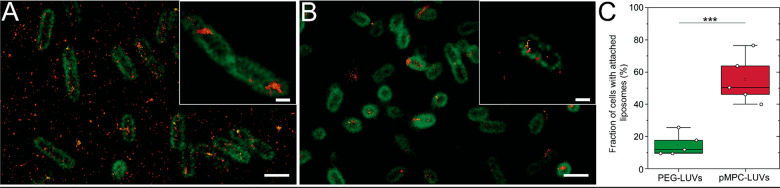
Representative super-resolution microscopy images of PA14 cells after 4 h incubation with (A) pMPCylated and (B) PEGylated-liposomes at 37 °C. Inset shows a zoomed-in detail of bacteria cells with liposomes adhering to the membrane. Images are presented as overlays of bacteria membrane stained with FM1–43 dye (green) with liposomes labeled with DiR dye (red) visualized using stochastic optical reconstruction microscopy (STORM). Scale bar, 1 μm; insert scale bar 1 μm. (C) Quantification of bacteria cells containing liposomal fluorescence from STORM images. Each fluorescent locus on the cells’ membrane with a minimum size of 180 nm was counted as one individual cell-attached liposomal unit. Differences between groups shown in the box plot were tested with a one-way ANOVA. Boxes represent the 25–75 percentiles of the sample distribution, with black vertical lines representing 1.5 × IQR (interquartile range). Black horizontal line represents the median.

Super-resolution microscopy (STORM) at a single-cell level was used to examine the distribution of pMPCylated liposomes following their incubation for 4 h with *P. aeruginosa* cells. We found that pMPCylated LUVs (Figure 3A) bound ∼3.3-fold more to the outer bacterial membranes than PEGylated LUVs used as a control (Figure 3B,C). A significant number of pMPCylated vesicles are also observed in the intercellular space (Figure 3A). As cells were thoroughly washed following their incubation with the liposomes, and only then placed on the imaging glass, it is clear that these LUVs are not attached to the glass substrate. Rather, they may be adhering either to the residual extracellular matrix and/or to filamentous structures extending from the PA14 cells.^37,38^

### pMPCylated-Liposomes Efficiently Deliver Cargo into the Bacterial Cytosol

**Figure 4:**
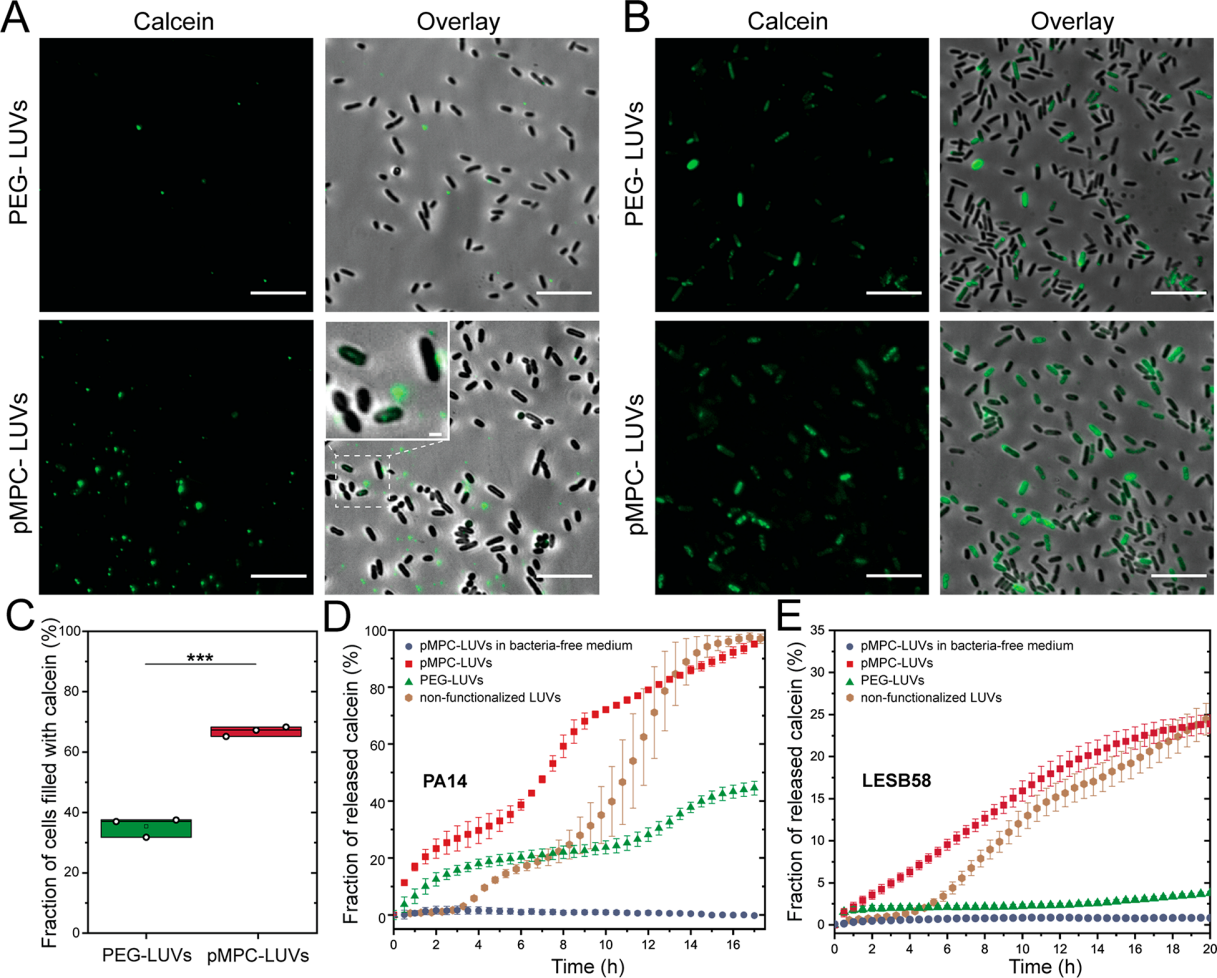
Internalization and cargo release by calcein-encapsulating PEG- and pMPC-functionalized vesicles incubated with *P. aeruginosa* cells and biofilms. Representative microscopy images of PA14 cells following 4 h (A) and 24 h (B) incubation, followed by thorough washing, with PEG- (upper panels) and pMPC- (lower panels) calcein-loaded liposomes. Cells were grown at 37 °C for 24 h prior to the incubation with liposomes. Images are presented as a fluorescent intensity of the calcein signal (green) and overlay between calcein-fluorescence and brightfield. Insert in lower right panel (A) shows a zoomed-in detail of bacteria cells displaying a weak but recognizable fluorescent intensity. Scale bars: main, 10 μm; insert, 1 μm. (C) Quantification of single cell microscopy images of the number of cells displaying luminal calcein signal following 24 h incubation of cells with either PEG-LUVs or pMPC-LUVs. Differences between groups shown in the box plot were tested with a one-way ANOVA. Boxes represent the 25–75 percentiles of the sample distribution, with black vertical lines representing the 1.5 × IQR (interquartile range). Black horizontal line represents the median. (D) and (E) Kinetic profile of calcein release from liposomes upon interaction with bacterial biofilms of strain PA14 (D) or LESB58 (E) at 37 °C incubated for 17 h. Liposomes were either nonfunctionalized (brown circles) or functionalized with 5% pMPC (red squares) and 5% PEG (green triangles) polymer. Calcein-loaded pMPC- LUVs were incubated with naive BM2G media as a negative control (blue circles). Results are an average of a minimum of three experiments.

The delivery efficiency of pMPCylation in releasing liposomal cargo into the bacteria cytosol was determined with single-cell fluorescence microscopy imaging of bacteria incubated with calcein-loaded liposomes at *t* = 4 h and at *t* = 24 h, as shown in Figure 4, following thorough washing of the cells after incubation. The 4 h incubation reveals, in agreement with the STORM images (Figure 3A), a higher number of pMPC-functionalized liposomes compared to PEGylated LUVs, with small lipid aggregates visible both near the bacteria membrane and in the intercellular space (Figure 4A lower panel, especially lower right inset). Following 24 h, pMPCylated LUVs released their calcein cargo into a much larger fraction of bacteria (70%) compared to PEGylated liposomes (35%) (Figure 4B,C), and moreover with a brighter calcein signal per cell. Interestingly, after 24 h we do not observe pMPC liposomes in the intercellular spaces (Figure 4B), suggesting that most of the added vesicles have been internalized by cells.

The kinetics of the observed payload delivery was studied via calcein dequenching assay,^39^ and is shown in Figure 4D,E for two different strains of *P. aeruginosa*. The improved efficiency of pMPCylated liposomes compared to PEGylated LUVs is clearly seen, with nearly 100% of the total dye being released by the former over 17 h as opposed to ca. 40% for the latter (for the PA14 strain, Figure 4D). This 2.5-fold difference in cargo release arises entirely from the different interactions between the respective liposomes and the biofilm, as incubation of liposomes with bacteria culture media (BM2G) alone shows no changes in calcein intensity (Figure 4D and Figure S7) or liposome size (Table S2). Moreover, pMPCylated vesicles undergo faster interaction and cargo release than nonfunctionalized liposomes; while both conditions achieve full delivery within 17 h, the nonfunctionalized LUVs show a 4 h lag time prior to any calcein release. Additionally, such nonfunctionalized vesicles are unstable and aggregate within 3 days (Figures S5 and S6). To examine the generality of these observations, we repeated these measurements in a *P. aeruginosa* clinical strain: LESB58, a hypervirulent human cystic fibrosis isolate,^40^ as shown in Figure 4E. While the overall release efficiency in this specific strain is, for both functionalizations, lower compared to PA14, with 25% release of the total calcein for pMPC-LUVs, nonetheless the fold-differences are even larger, with pMPCylated vesicles having ca. 5-fold higher cargo release into the LESB58 biofilm compared to PEGylated ones. The variation in total cargo release (100% for PA14 vs 25% for LESB58 following 20 h) may be due to differences in lipid^41,42^ and biomolecules composition at the different bacterial surfaces.^43^

Based on these results obtained using a combination of cryo-TEM, STORM and confocal microscopy, and fluorimetry (Figures 1–4), a two-step mechanism emerges for the interaction between pMPC-stabilized liposomes and bacterial membranes. Functionalization with pMPC promotes liposome–bacteria adhesion via divalent-ions-induced LPS bridging (which is not the case with PEG-LUVs). This is schematically illustrated in Figure 7 and discussed in more detail below (pMPCylated Carriers May Be Efficiently Loaded with Multiple Drug Agents). This adhesion leads to a higher fusion rate,^44^ with subsequent release of the vesicles cargo into the bacterial cytosol, as measured via single-cell microscopy and calcein dequenching technique (see also pMPCylated Carriers May Be Efficiently Loaded with Multiple Drug Agents and Conclusions).

### pMPCylated Carriers May Be Efficiently Loaded with Multiple Drug Agents

**Figure 5:**
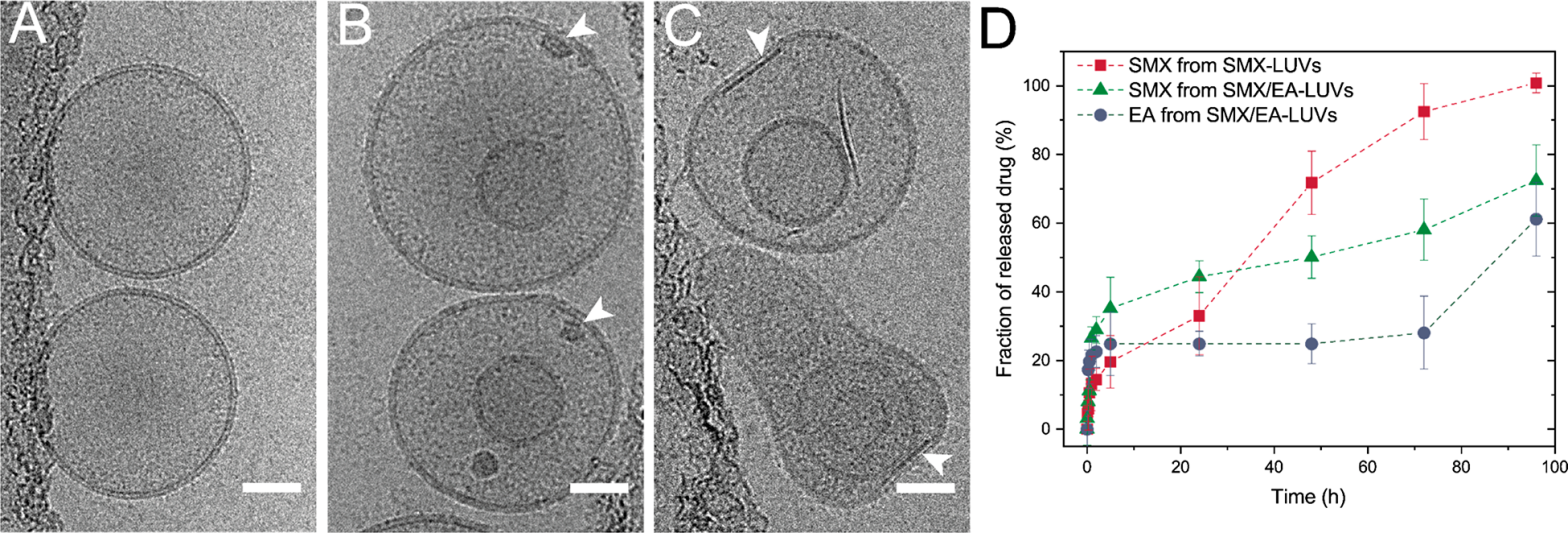
Representative cryo-TEM images of pMPCylated vesicles: (A) unloaded liposomes, (B) liposomes loaded with SMX, and (C) coloaded with SMX/EA. White arrows indicate a dark structure inside liposomes. Scale bar, 50 nm. (D) Release profiles of SMX, EA, and SMX/EA from pMPC-functionalized liposomes at physiological conditions (37 °C, pH 7.2). Results are shown as an average and standard deviation of three independent experiments. Dashed lines represent a trend of the data points.

Liposomes were loaded either with the antibiotic sulfamethoxazole (SMX), or coloaded SMX with the phytochemical ellagic acid (EA, Table S3).^45^ EA has been shown to downregulate gene expression in bacteria^46^ and suppress pyocyanin production in *P. aeruginosa* strains (Figure S14),^47^ resulting in enhanced cell sensitivity toward antimicrobial agents, as previously demonstrated for SMX.^48^ The two compounds were sequentially encapsulated (EA then SMX) within the liposomes using a modified transmembrane gradient approach (Methods section),^49^ resulting in a green-tinted sample (Figure S8). The final SMX:EA:lipid molar ratio was 0.22:0.6:1, a 60% lower antibiotic dosage compared to SMX-only liposomes, where maximal encapsulation was at a SMX:lipid molar ratio of 0.55:1.0. Successful encapsulation was visualized with Cryo-TEM imaging (Figure 5), revealing the presence of both SMX and EA inside liposomes as darker interior, with long elongated crystals altering the vesicles’ shape (Figure 5C and Figure S9B,C). Conversely, unloaded liposomes appear unaltered (Figure 5A), whereas SMX-only loaded LUVs lack crystal features and present only darker structures (Figure 5B and Figure S9A). Despite shape alteration, the overall SMX-EA-LUV size distribution displays a negligible 10 nm diameter increase upon loading, independently of surface functionalization (Table S1), and comparable for both loading configurations.

Release profiles of the loaded pMPCylated liposomes at physiological conditions (37 °C, pH 7.2) show that SMX alone is fully (100%) released over 100 h, while SMX or EA from coloaded vesicles has slower release kinetics, with only ca. 60% of the total encapsulated drug being released over the same period. PEGylated liposomes have similar release profiles (Figure S10). The stability of the encapsulated compounds shows long-term storage at 4 °C (up to 3 months) retaining 90 ± 5% of the drug (Figure S11).

### pMPCylation markedly increases eradication of bacterial biofilms

**Figure 6:**
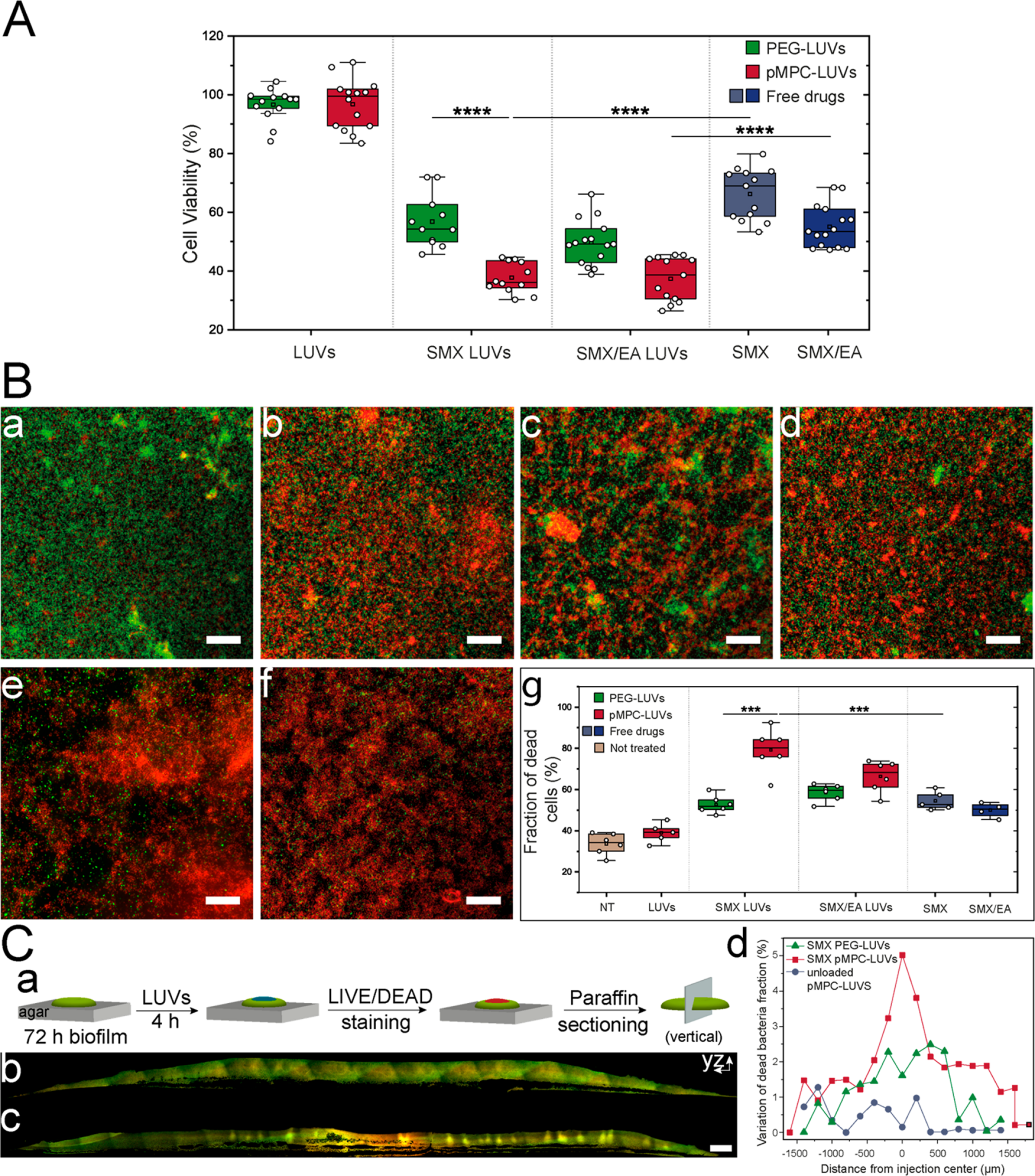
Effect of SMX and SMX/EA-LUVs on biofilm viability. (**A**) PA14-cell viability after two-dose treatment (4h each) with SMX- and SMX/EA- LUVs and free drugs quantified by MBEC-based resazurin assay. Blue boxes (SMX [1.5 mg/mL], SMX/EA [0.7/1.5 mg/mL]) represent treatment with free drugs. Data obtained from 8 or more biological repeats. (B(a–g)) Representative confocal microscopy images of the antibacterial effect of drug-loaded LUVs and free drugs on 24 h biofilm evaluated by a live (green)/dead (red) assay. Images show representative areas from chamber slides. (a) nontreated (NT) biofilm; (b) free SMX [1.5 mg/mL]; (c) SMX-loaded PEG-LUVs; (d) SMX/EA-loaded PEG-LUVs; (e) SMX/EA-loaded pMPC-LUVs; (f) SMX-loaded pMPC-LUVs. Scale bar, 50 μm. (g) Percentage of dead bacteria quantified from at least four different microscopic images. Data represent a minimum of two biological repeats with two technical repeats each. (C(a–d)): Confocal microscopy of cross sections of paraffin-embedded PA14-colony after 4h treatment with pMPC-LUVs loaded with SMX. (a) Preparation of a 10 μm-thin section of the colony upon treatment with (b) 5 μL unloaded pMPC-LUVs or (c) 5 μL pMPC-LUVs loaded with SMX. Live (green)/dead (red) staining was applied prior to fixation. Scale bar, 100 μm. (d) Spatial profiles for biofilm sections displaying variation in dead bacteria cells fraction upon injection with either SMX-loaded pMPC-LUVs (red) or PEG-LUVs (green), or nonloaded liposomes (blue). Details of the quantification in the Methods sections and Figure S15. Differences between groups shown in box plots were tested with a one-way ANOVA. Boxes represent the 25–75 percentiles of the sample distribution, with black vertical lines representing the 1.5 × IQR. Black horizontal line represents the median.

Eradication of *P. aeruginosa* biofilms (PA14 strain) was examined following two 4 h of consecutive administrations of drug-loaded liposomes using a MBEC-based resazurin assay (see Methods section), and results are shown in Figure 6A. Incubation with control nonloaded liposomes shows no significant changes in cell viability compared to untreated cells. Delivery of coloaded SMX-EA- or SMX-loaded LUVs results in a clear loss of cell viability, with larger loss for coloaded carriers. pMPCylated-LUV carriers achieved 60–65% viability reduction compared to nontreated bacteria, significantly better than PEGylated-LUVs (45–50% reduction) and even more significant compared to free-drug treatment (30–45% viability reduction). The improved effect with SMX-EA compared to SMX-alone is especially notable as the actual encapsulated amount of antibiotic in the coloaded LUVs is reduced by 60%, indicating that EA strongly enhances SMX effectiveness, as also seen when converted to colony-forming units (CFU) (Figures S12 and S13).

To complement results from resazurin spectra, which are dependent on bacterial metabolism and report only live bacteria, we also directly imaged and quantified the fraction of dead bacteria in the biofilm population following treatment with the different liposomal configurations and with free antibiotics. Visualization of PA14 biofilms via live/dead fluorescence staining and confocal microscopy is shown in Figures 6B(a–g) and approximately mirrors the findings obtained by MBEC -resazurin assay (Figure 6A). Untreated biofilms incubated with empty liposomes show a predominant green (live) signal (Figure 6B(a)), whereas treatment with free SMX produces a significant increase in the number of red loci, i.e., dead cells (Figure 6B(b)). Treatment with two doses of either SMX-EA or SMX- LUVs results in more dead bacteria, for both functionalizations, with pMPCylation displaying the highest biofilm eradication, up to 85%, compared to PEGylation (50–60% dead cells) or to free drugs (50–55%) (Figure 6B(g)).

**Figure 7:**
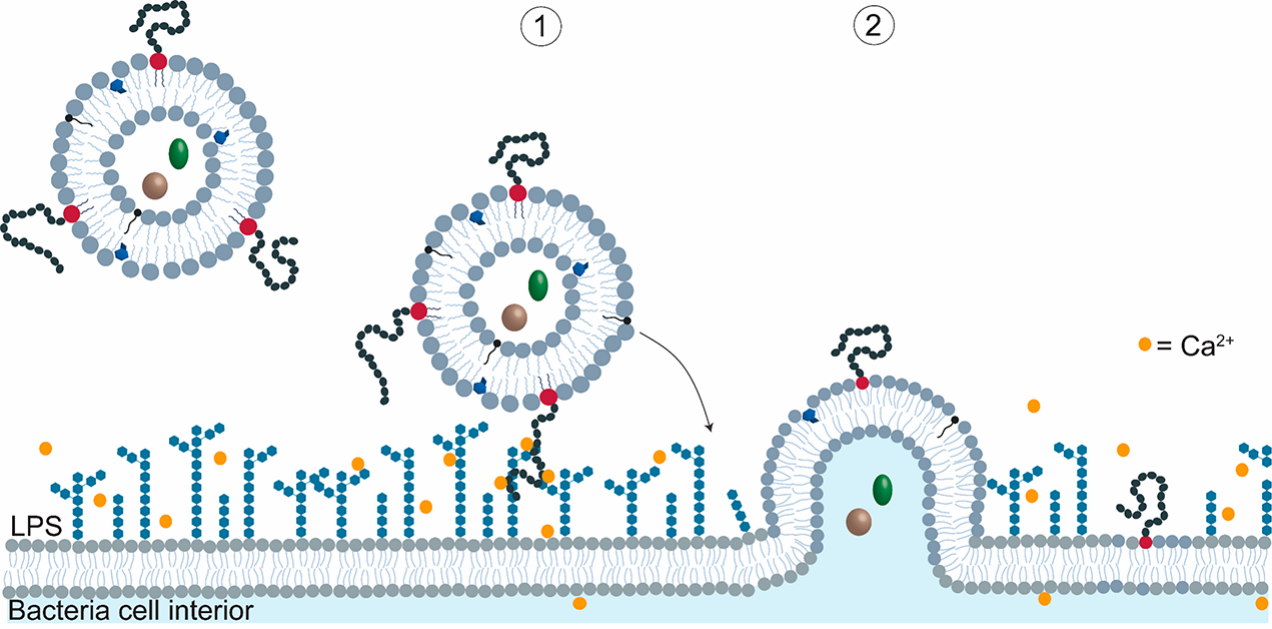
Schematic of proposed two-stage mechanism for calcium-mediated adhesion and fusion between pMPC- LUVs and *P. aeruginosa* membrane. (1) Liposomes functionalized with pMPC reach the bacterial membrane, where the presence of divalent cations (yellow points) at the cell membrane’s surface bridges pMPC with LPS and pulls them toward the cell surface. (2) Fusion between liposome and bacterial membrane occurs due to charge–charge interaction (see text).

Finally, as a proof of concept, we employed the live/dead confocal microscopy assay on biofilm colonies grown on a solid medium to demonstrate that pMPCylated liposomes can readily deliver their cargo even to an air-exposed biofilm, commonly found in wound and lung infections as well as nonbiological surfaces.^50,51^*P. Aeruginosa* (PA14 strain) biofilm colonies were grown and embedded in paraffin post-treatment with liposomes vehicles, to enable coronal sectioning and subsequent microscopy imaging (Figure 6C(a)). Treatment with nonloaded LUVs shows a physiological fraction of coexisting dead (red signal) and live bacteria (green signal, Figure 6C(b)). The addition of SMX-loaded liposomes, followed by 4 h incubation, results in a clear increase of dead bacteria (red) at the point of injection (Figure 6C(c)), demonstrating that pMPC-functionalized liposomes may also be used to treat air-exposed biofilms too. Image quantification (Figure 6C(d) and Methods section) confirms the antimicrobial effects along a gradient from the injection point and shows that pMPCylated-LUVs achieve a 2.5-fold higher eradication of bacteria relative to PEGylated-LUVs (Figure S15), highlighting their higher delivery efficiency even in challenging conditions like administration to air-exposed biofilms.

## Conclusions

The main findings of this study are that a pMPC functionalization of liposomal drug delivery vehicles results in significantly higher biofilm penetration, cell interaction, and bacterial eradication than equivalent free drug or current liposome-based treatments of such biofilms. This is due to a combination of several beneficial effects arising from the pMPCylation, as considered below.

The pMPC functionalization of liposomes improves targeting toward bacteria cell membranes with 4-fold higher affinity compared to other functionalization strategies (PEG) (Figure 3) and, as a result of membrane adhesion, liposomes fuse with bacteria cells with much higher efficiency (Figures 4A–C). We demonstrate that the observed enhanced fusion results in higher cargo release into bacteria cytosol than other functionalized counterparts: between 2.5-fold and 5-fold faster for the two bacterial strains examined, as shown in Figure 4D,E respectively. The enhanced cytosolic release observed for both strains indicates the potential of our carriers to be effective over a broad range of biofilm infections. We attribute this significantly more efficient performance of the pMPCylated nanocarriers to the structure of the functionalizing moieties. The monomers of the pMPC oligomers, whose structure resembles the phosphocholine headgroups of PC lipids, the most common lipid type, have a strong dipole arising from their dual charge nature (Figure 1A). In the presence of divalent ions, which (mainly in the form of Ca^2+^) are physiologically ubiquitous,^52^ these dipoles can strongly interact with cells, as discussed below. This is indicated in Figure 1B, where the presence of Ca^2+^ ions leads to a clear attraction between the pMPCylated vesicles arising from divalent-ion bridging of the pMPC moieties (Figure 1C).

The stronger interaction of the pMPCylated LUVs with the target cells in this study arises, as observed in vitro using calcein dequenching measurement of bacterial-mimicking LUVs (Figure 2B), from the bridging of the MPC monomers to the negatively charged LPS exposed at the cells’ surface via divalent cations present at the bacterial membrane.^25,53^ This may be described by the two-stage mechanism noted earlier (as shown schematically in Figure 7), where pMPC modulates the liposome-cell adhesion. In the first stage, increasing overlap of the pMPC moieties with the surface-exposed negatively charged LPS at the bacterial outer membrane is energetically favored by their Ca^2+^-mediated interactions. Maximizing this overlap results in the pMPCylated liposomes being “pulled in” toward the cells and to proximity-driven fusion between the liposome and cell lipid membranes, which is the second stage. We attribute such fusion to the charge–charge interactions (the positively charged SA with the negatively charged bacterial membrane), as previously reported.^43^ We note that while the presence of negatively charged LPS at the surface of the (Gram-negative bacteria) target cells suggests this specific bridging configuration in the present study, similar divalent-ion mediated interactions would be expected between the dipolar MPC monomers and different negatively charged groups also present at other cell surfaces.^54^ It would be therefore of interest in future work to examine whether pMPCylated liposomes exhibit enhanced uptake also by nonbacterial cells.

As a direct consequence of the enhanced interaction mechanism, pMPCylated liposomes are significantly more efficient in killing target cells in bacterial biofilms, compared to other liposomal carriers (PEG-functionalized) tested in our study or to free compounds, as seen clearly in Figure 6B(g). At the same time, pMPCylated vesicles are fully equivalent to PEG-functionalized ones regarding their biocompatibility (Figure 6A) and their long-term colloidal stability (Figure S1).

Finally, we demonstrate the drug-loading versatility of our pMPC-liposomes, as they can efficiently and sustainably load single compounds (e.g., the antimicrobial agent SMX) or multiple agents simultaneously (SMX and the performance-enhancing phytochemical EA). This therapeutic approach relying on multiple drug combinations results in decreased toxicity of bacterial treatment by employing a lower concentration of antibiotics and enhancing their antimicrobial activity by synergistic interplay (Figure 6).^55^

In summary, our pMPC-based liposomal functionalization strategy exhibits enhanced penetration and delivery efficiency to bacterial biofilms, including different strains and both wet and air-exposed biofilms, as well as multidrug loadings to improve therapeutic ability. Such properties are particularly attractive for applications in clinical settings, where the vast majority of biofilm-based bacterial infections occur. The major hurdle in such occurrences is that biofilms are not easily targeted by existing antibiotic-delivery systems due to difficulties in penetrating the films physical barriers and overcoming drug resistances.^56,57^ This results in currently available treatments available for biofilm infections being poorly efficient and very side-effects prone.^50^ Thus, the enhanced bacterial affinity and penetration of pMPC carriers would potentially provide better outcomes for patients (more efficient eradication and corresponding quicker recovery) and lower adversary effects (lower drug dosage). Moreover, the multidrug loading approach could be employed to target drug-resistant strains. All these benefits could apply to multiple potential scenarios, including not only topical wound infections^50^ but also infection at biomedical devices interfaces,^58^ post-transplant care,^59^ and inhalation-based strategies for respiratory infections,^60^ with significant benefits for a multitude of patients. We believe these transformative features have the potential to lead to the next generation of liposomal delivery systems for bacterial infections and to be successfully translated to clinical settings.

## Methods

### Materials

Hydrogenated soybean phosphatidylcholine (HSPC, Mw 786.11), cholesterol (C_27_H_46_O, Mw 386.65), stearylamine (CH_3_(CH_2_)_17_NH_2_, Mw 269.509), 18:0 PEG5000 PE (1,2-distearoyl-*sn*-glycero-3-phosphoethanolamine-*N*-[methoxy(polyethylene glycol)-5000] (ammonium salt), Mw 5801.071), *Escherichia coli* extract total (100500P), lipopolysaccharides from *E. coli* O111:B4 (L2630), magnesium acetate ((CH_3_COO)_2_Mg, Mw 142.39), sodium sulfate (Na_2_SO_4_, Mw 142.04), calcein (C_30_H_26_N_2_O_13_, Mw 622.53), sulfamethoxazole (C_10_H_11_N_3_O_3_S, Mw 253.28) and Resazurin (C_12_H_6_NNaO_4_, Mw 251.17), l-lysine hydrochloride (H_2_NCH_2_(CD_2_)_2_CH_2_CH(NH_2_)CO_2_H·HCl, Mw 186.67), Sephadex G-25 in PD-10 Desalting Columns (GE Life Science), and dialysis bag - Float-A-Lyzers were purchased from Sigma-Aldrich (Israel). Ellagic acid (EA, C_14_H_6_O_8_, Mw 302.197) was purchased from Carbosynth Limited (Compton, UK). DiI Stain (1,10-dioctadecyl-3,3,30,30-etramethylindocarbocyanine perchlorate, C_59_H_97_ClN_2_O_4_, Mw 933.88), HPTS dye (8-hydroxypyrene-1,3,6-trisulfonic acid, trisodium salt, C_16_H_7_Na_3_O_10_S_3_, Mw 524.37), and LIVE/DEAD BacLight Bacterial Viability Kit (C_27_H_34_I_2_N_4_, Mw 668.4) were provided by ThermoFisher Scientific (Waltham, MA, USA). All chemicals had high purity and were used without further purification. Cell Proliferation Kit (XTT based) was purchased from Biological Industries (Israel Beit Haemek LTD, Israel). MBEC Biofilm Technologies Ltd. Calgary, AB, Canada. HPLC-grade water was obtained from J.T. Baker. HPLC grade acetonitrile, 5-methyl-1(5H)-phenazinone (Pyocyanin), trifluoroacetic acid (TFA), and 1,5-naphthalenediamine were purchased from Merck. HCl was purchased from BioLab.

### Liposome Preparation (LUVs), Size, and ζ-Potential Characterization

HSPC/cholesterol (40% mol/mol)/pMPC (5 kDa, 5% mol/mol) or HSPC/cholesterol (40% mol/mol)/PEG (5 kDa, 5% mol/mol) or unfunctionalized liposomes composed of HSPC/cholesterol (40% mol/mol) were dissolved in chloroform or chloroform/methanol mixture (2:1), and organic solvent was evaporated using first nitrogen stream, followed by 8 h of vacuum pumping. For introducing a positive charge into liposomes, 5% (mol/mol) of stearylamine was added to chloroform before evaporation. The lipid film was then hydrated with an aqueous solution of (CH_3_COO)_2_Mg (140 mM, osmol = 320 mOsmo/kg) at 70 °C to reach the desired concentration and solution was gently vortexed. The resulting multilamellar vesicles (MLVs) suspensions were sonicated for 15 min at 70 °C to disperse larger aggregates. The vesicles were subsequently downsized by extrusion (Lipex, Northren Lipids Inc.) through 400, 200, and 100 nm polycarbonate membranes. The extrusion was performed 11 times through each membrane at 65 °C.

Bacteria-mimicking liposomes: *E. coli* extract was dissolved in a mixture of chloroform and methanol (2:1 v:v) and evaporated using the first nitrogen stream, followed by 3 h of vacuum pumping. For LPS -pMPC interaction studies, an additional *E. coli* extract mixed with 32 ng/mL of LPS was prepared in an organic solvent. The lipid film was then hydrated with 5 mL of calcein solution (35 mM, osmol = 300 mOsmo/kg) at 35 °C. The resulting MLVs suspension was sonicated for 15 min at 35 °C and subsequently downsized by extrusion (Lipex, Northren Lipids Inc.) through 400 nm and 200 nm polycarbonate membranes. The extrusion was performed 11 times through each membrane at 35 °C. Liposomes were then separated from an excess of free calcein by 48 h - dialysis (Float-A-Lyzers, Sigma-Aldrich) against PBS.

The size and ζ-potential of the lipid nanoparticles were measured with a ZetaSizer Nano ZS (Malvern Instruments, UK) at 25 °C. Triplicate measurements with a minimum of 10 runs were performed for each sample.

### Calcein Assay

Membrane fusion of surface-functionalized (either with PEG- or pMPC-) or bare liposomes with bacteria biofilm or with bacteria-mimicking liposomes was monitored by calcein dequenching methods as described elsewhere.^39^

(1) Functionalized or bare liposomes were prepared using an extruder, where lipid film was hydrated with a solution of (CH_3_COO)_2_Mg containing 70 mM calcein. Osmolarity of (CH_3_COO)_2_Mg/calcein mixture was adjusted to 320 mOsmo/kg. The loaded vesicles were then separated from an excess of free calcein by Sephadex G-25 preequilibrated with Na_2_SO_4_ followed by 24 h of dialysis (Float-A-Lyzers, Sigma-Aldrich) against Na_2_SO_4_. The calcein-loaded liposomes (20 μL, 2 mM final concentration, shaken) were added to 150 μL of 24 h-biofilm.
(2) Bacteria-mimicking liposomes (*E. coli* total extract) were prepared using an extruder, where lipid film was hydrated with calcein solution (35 mM, osmol = 300 mOsmo/kg). Twenty-five μL of bacteria-LUVs (concentration 1.7 mM) was added into 20 μL of 10 mM HSPC/cholesterol (40% mol/mol)/pMPC (5 kDa, 5% mol/mol) and 160 μL PBS (−/– Ca^2+^/Mg^2+^) or PBS (+/+ Ca^2+^/Mg^2+^).

Continuous monitoring of calcein fluorescence (excitation 470 nm, emission 509 nm) was done at intervals of 15 min for a period of 24 h (without shaking) at 37 °C, using a ClarioStar microplate reader (BMG LABTECH GmbH, Germany). The final fluorescence intensity, which represents maximal fluorescence of free calcein, was determined following the solubilization of vesicles with Triton X-100 (2% v/v) and compared to the value of calcein from solubilized liposomes in the biofilm- or bacteria-liposome free medium.

### Giant Unilamellar Vesicles Preparation

Giant unilamellar vesicles (GUVs) of DPPC/pMPC (5 kDa, 5% mol/mol) or DPPC/PEG (5 kDa, 5% mol/mol) labeled with 0.1% (mol/mol) DiI dye were prepared using the poly(vinyl alcohol) (PVA) gel-assisted formation method as described by Weinberger et al.^61^ Briefly, 200 mL of 5% (w/w) PVA solution was spread on a glass slide and dried for 30 min at 80 °C. Once the PVA-coated substrate was prepared, 5 mL of lipid in chloroform/methanol (2:1) (1 mg/mL) was spread and placed under vacuum for 30 min to evaporate the organic solvent. Using a rubber gasket as a temporary chamber, the lipid film was hydrated with a PBS solution (318 mOsm/kg) and left incubating for 60 min at 50 °C. After incubation, the GUVs were collected and transferred to microscopy glass.

### Remote Drug Loading and Efficiency Determination

Active loading of SMX and SMX/EA was based on a modified approach of Clerc et al.^49^ Freshly prepared lipid suspension was passed through a size-extrusion column, Sephadex G-25, preequilibrated with Na_2_SO_4_ (pH ∼ 5.5, 320 mOsmo/kg), which creates a proton/transmembrane gradient inside the lipid carrier. The low pH outside of the vesicles allows the drug to be uncharged and therefore to freely diffuse across the lipid membrane toward the inside. The higher pH of the liposomes’ lumen allows ionization of the drug, favoring its accumulation. Liposomal sample was heated above phase transition of HSPC-lipid (65 °C), and a defined amount of SMX in DMSO (5% v/v) was added. Magnetic stirring allowed a homogeneous distribution of drug in sample. For single-drug-loaded liposomes, the sample was left for 90 min at 65 °C. For liposomes loaded with both SMX and EA, after 90 min of equilibration with SMX, EA was added at a concentration 1.1 mg/mL, and the sample was equilibrated for another 45 min. Subsequently, samples were cooled rapidly in an ice bath, and non-entrapped drugs were removed using a size-extrusion Sephadex G-25 column preequilibrated with BM2G or Na_2_SO_4_ (pH 7.0, ∼320 mOsmo/kg). To ensure complete removal of residual DMSO, samples were overnight dialyzed (MWCO 50 kDa, 4 °C) against BM2G or Na_2_SO_4_ (pH 7.0, ∼320 mOsmo/kg).

To measure intraliposomal drug content after loading, 20 μL of the sample was diluted with 1 mL of ethanol (for EA determination, 10 mM Borax was added to the solution) and placed in a bath sonicator for 10 min. The concentration of SMX and EA inside liposomes was determined by an ultraviolet spectrophotometer (Cary 100 Bio, Varian Inc., USA) at wavelengths 264 and 367 nm, respectively. The loading efficiency was calculated as a mole ratio between loaded drug and HSPC lipid. Concentration of HSPC lipid after the loading procedure was determined based on Nanosight (NTA) results.

For NTA measurements, samples were diluted with PBS to a final volume of 1 mL. Optimal dilution for each sample was found by pretesting the sample until ideal particle-per-frame value (20–100 particles/frame) was obtained. For each measurement, five 1 min videos were captured at 25 °C, with at least 300 μL displacement between each video. The number of completed tracks in NTA (Malvern NanoSight NS300, Malvern, UK) measurements was always greater than the proposed minimum of 1000 in order to minimize data skewing based on single large particles.

### Determination of Drug Release Profile

Drug-loaded LUVs sample was placed in a dialysis tubing with a molecular cutoff of 50 kDa, and the sample was dialyzed against PBS buffer (∼320 mOsmo/kg, pH 7.4). Sample was incubated at 37 °C, and at defined time points, 20 μL of sample was collected and analyzed for drug content as described above, and PBS outside the dialysis bag was exchanged.

### Cryo-TEM Measurements

A Vitrobot plunger system Mark IV (FEI, USA) was used to prepare the samples for cryo-TEM. Humidity was kept close to 80% for all experiments, and the temperature was set at 24 °C. Three μL of the liposomal suspension (concentration, 1.5 mg/mL) was placed onto a holey carbon grid (C-flat 2/2 200 mesh) (Electron Microscopy Sciences, Hatfield, PA, USA) which was rendered hydrophilic via glow discharge (30 s, 25 mA) (PELCO easiGlow, Redding, CA, USA). Excess sample was removed by blotting (3 s) with filter paper, and the sample grid was vitrified by rapid plunging into liquid ethane. The sample imaging was conducted on a Talos Arctica G3 TEM/STEM (FEI, USA) cryo-electron microscope equipped with a OneView camera (GATAN) at an accelerating voltage of 200 kV. Images were acquired in low-dose mode using the SerialEM software (FEI, USA) to avoid radiation damage to the samples at 73000× magnification with a defocus value in the range of [−2 μm; −4 μm].

### General bacterial growth conditions

*P. aeruginosa* UCBPP-PA14 (1) or *P. aeruginosa* strain LESB58 (an epidemic strain isolated from chronically infected CF patients) precultures cultures were inoculated at OD_600_ = 0.05 from an overnight culture, and growth was carried out at 37 °C with shaking for 4 h, in Luria–Bertani broth (LB) for PA14 and tryptic soy broth (TSB) medium for LESB58 until midexponential phase (OD_600_ ∼ 0.5). Then to form a biofilm, bacteria were diluted 1:100 in BM2G minimal medium [62 mM potassium phosphate buffer, pH 7, 7 mM (NH_4_)_2_SO_4_, 2 mM MgSO_4_, 10 μM FeSO_4_, 0.4% (wt/vol) glucose, 1% monosodium glutamate] and grew in a 96-well plate at 37 °C without shaking for 24 h.^62^

For CFU, bacteria were grown in BM2 at 37 °C with shaking for 16 h every 2 h, 100 μL of bacteria was serially diluted in sterile PBS, and the dilutions were plated on two LB agar medium. Total bacterial counts were obtained and compared to the Resazurin assay.

### Single Cells Fluorescence Microscopy

Liposomal fusion with bacteria cells was visualized using single cell fluorescence microscopy. In brief, LUVs were prepared using an extruder, and the lipid film was hydrated with a solution of magnesium acetate containing 70 mM calcein. The osmolarity of the magnesium acetate/calcein mixture was adjusted to 320 mOsmo/kg. The calcein-loaded vesicles were then separated from an excess of free calcein by Sephadex G-25 pre-equilibrated with BM2G medium (∼320 mOsmo/kg) followed by overnight dialysis (MWCO 50 kDa) against BM2G.

PA14 cells were grown in BM2G medium at 37 °C, 200 rpm. When growth reached an OD_600_ = 0.3, bacteria were incubated with liposomes loaded with calcein (0.03 mM final liposomal concentration) for 24 h at 37 °C. At *t* = 4 h and *t* = 24 h, 500 μL of the samples was centrifuged at 8000*g* for 2 min at 25 °C and resuspended in 10 μL PBS. Samples were visualized using an Axioplan2 microscope (ZEISS, Germany) equipped with ORCA Flash 4.0 camera (HAMAMATSU). System control and image processing were performed using Zen version 2.6 (Zeiss, Germany).

### Super-Resolution Microscopy

Three-dimensional d-STORM imaging was performed using Vutara SR352 microscope (Bruker, USA) based on single-molecule localization biplane technology. Images were recorded using 1.3 NA 60× silicon oil immersion objective (Olympus) and Hamamatsu Orca Flash 4v2 camera with a frame rate at 50 Hz. Bacteria were incubated with liposomes labeled with DiR (0.5% mol/mol) dye for 4 h, then washed three with PBS. Next, bacteria were stained with 1 mg/mL membrane stain FM1-43 (Thermo Fisher Scientific T35356), washed three times with PBS, centrifuged at 5000 rpm for 2 min at 25 °C, resuspended. The bacteria were fixed with 2.8% formaldehyde (FA), 0.04% glutaraldehyde-formaldehyde (GA) for 15 min, washed three times, centrifuged at 5000 rpm for 2 min at 25 °C, and resuspended in 10 μL PBS. Bacteria were dropped on a poly-l-lysine (Sigma P8920) coated MatTek plate (P35G-1.5-7-C), and imaging was performed in the presence of an imaging buffer (7 μM glucose oxidase (Sigma), 56 nM catalase (Sigma), 2 mM cysteamine (Sigma), 50 mM Tris, 10 mM NaCl, 10% glucose, pH 8). Subsequently, STORM images of liposomes were recorded using 640 nm excitation laser (maximal excitation of 6 kW/cm^2^) with a collection of 3000 frames. Data were analyzed with the Vutara SRX 7.0.00rc24 software.

### Determination of Biofilm Eradication by a Combination of MBEC and Resazurin Assays

PA14-biofilms were formed using MBEC assay system (Innovotech, USA) as previously described with slight modifications.^63^ Briefly, bacterial suspension was adjusted to OD ∼ 0.5. The biofilm device was inoculated by adding 150 μL of the inoculum into the wells of the 96 peg-lids on which the biofilm cells could build up. Respective negative control (BM2G medium only) wells were also prepared. The pegs were incubated in a humidified incubator for 24 h at 37 °C to allow biofilm formation on the purpose-designed pegs. Once biofilm was fully formed, the PEG-lid was washed by moving for 10 s to a new 96 well containing 160 μL of PBS in each well. The washing procedure was repeated two times. Subsequently, the peg lid was then transferred into a ‘challenge 96-well microtiter plate’ containing 170 μL of antimicrobial treatment, i.e., free SMX, or liposomes loaded with antimicrobial treatment. The peg lid was incubated for 4 h at 37 °C. After incubation, the peg lid was washed with 180 μL of PBS, and once more the biofilm was exposed to liposomal treatment. Finally, The peg lid was removed and washed twice with PBS as previously. PEG-lid was transferred into the recovery plate containing 190 μL of BM2G in each well and sonicated using a bath sonicator for 10 min at room temperature.

For cell viability, cells that were removed from the MBEC lid by sonication in BM2G medium containing 2 μL of resazurin stock solution (1 mg/mL) in a 96-well plate, and fluorescence (λ_ex_ 530 nm and 560–650 nm emission spectra) was using ClarioStar microplate reader (BMG LABTECH GmbH, Germany). Fluorescence signal was measured with 15 min intervals for 15 h at 37 °C with 100 rpm shaking mode. In the wells where no dye was added, the absorbance at 600 nm was measured.

### Determination of Bacterial Viability in Biofilms by LIVE/DEAD Staining

PA14- biofilms were developed on 8 well μ-Slide (ibidi GmbH, Germany). 150 μL of the inoculum was incubated in a humidified incubator for 24 h at 37 °C without shaking. After 24 h, the fully formed biofilm was gently washed twice with PBS. 160 μL of liposomal suspension or BM2G medium was added to each well, and subsequently, the samples were incubated at 37°C for 4 h. Each well was then washed with PBS, and a second dose (170 μL) of antibacterial treatment was applied for 4 h with incubation at 37°C. Subsequently, biofilm was washed with 180 μL of PBS buffer, and bacteria cells were stained using a BacLight Bacterial Viability Kit (ThermoFisher Scientific, USA). Live bacterial cells were stained with Syto 9 dye (ex: 486/em: 501), and dead cells were stained with PI dye (λ_ex_: 535/λ_em_: 617), with dyes mixed in 1:1 ratio. 170 μL of Syto9/PI in BM2G medium was added to wells and incubated for 20 min at room temperature. Later, wells were washed twice with PBS, and images of biofilm were acquired using confocal fluorescence microscopy. Cell viability quantification was performed using ImageJ software v1.52i (NIH, USA). Confocal pictures were acquired using a confocal laser scanning fluorescence microscope LSM700 (Zeiss, Germany). All images were acquired using a 40× oil immersion objective, with a 0.3 μm optical slice step for *z* scanning. Images were recorded in brightfield mode and in confocal mode using 488 excitation and 561 excitation laser channels. Picture analysis was performed using ImageJ software v1.52i (NIH, USA). For comparative analysis, all parameters during image acquisition were kept constant throughout each experiment.

### Paraffin-Embedded Thin Sectioning and LIVE/DEAD Staining for Fluorescence Imaging

Thin sectioning assays were performed as described in ref (64). Briefly, 5 μL of subcultures was spotted onto 1% agar plates containing a two-layered growth medium (1% tryptone, grown in the dark at 25 °C with >90% humidity). After 3 days, 10 μL of liposomes were added on the top of colony and incubated for 4 h in the dark at room temperature. Next, colonies were stained using 20 μL of a BacLight Bacterial Viability Kit (ThermoFisher Scientific, USA) for 15 min. Subsequently, colonies were covered with an agar layer, and sandwiched colonies were lifted from the bottom layer, washed for 10 min in PBS (pH 7.4) at room temperature in the dark, and fixed in 4% paraformaldehyde with 50 mM l-lysine hydrochloride in PBS overnight at room temperature in the dark. Fixed colonies were washed twice in PBS and dehydrated and paraffin-embedded through a series of ethanol washes (70%, 95% (×3), 100% (×2), ethanol/X-TRA SOLVE (MEDITE 41-5213-00) (50%/50%) for 1 h. Then, the colonies were paraffin-embedded via three 60 min incubations in X-TRA SOLVE 530 at 57 °C in fully enclosed tissue processor (Leicra ASP300S). Next, the colonies were allowed blocked overnight at 4 °C in Paraplast Plus Paraffin Wax (Leica Biosystems 39602004). Tissue processing was performed using an Tissue Embedding Medium Surgipath Paraplast Plus Paraffin White Solid. Trimmed blocks were taken in 1 mm deep from the center of the biofilm in the block, sectioned in 10 μm-thick sections, 5 μm angles perpendicular to the plane of the colony using microtome (Leica RM2265 Microtome), and collected onto slides. Slides were air-dried overnight, heat-fixed on a hot plate overnight at 37 °C, and rehydrated. Rehydrated colonies were immediately mounted in buffer (Leica Biosystems, EG 1160) and overlaid with a coverslip. Confocal pictures were acquired using a confocal laser scanning fluorescence microscope LSM700 (Zeiss, Germany). All images were acquired using a 40× oil immersion objective, and individual fields of view were subsequently stitched together to form the entire section. Images were recorded in brightfield mode and in confocal mode using a 488 excitation and 561 excitation laser channels. Picture analysis was performed using ImageJ software v1.52i (NIH, USA). For comparative analysis, all parameters during image acquisition were kept constant throughout each experiment.

### Data analysis

Images of bacterial slices displaying the full-length sample both in green and red channels were contoured to isolate only the biofilm section and remove the majority of the fluorescent background signal using Fiji. The resulting composite images with a removed background were then segmented with a 200 μm wide and 150–200 μm high region of interest to encompass the slice at its thickest point. For each segment, the average intensity of the two channels (green, LIVE signal; red, DEAD signal) was measured, and the fraction of dead bacteria was quantified as the ratio of the red intensity over the sum of the two channels. To compare different biofilm slices and different treatments, each spatial profile of dead bacteria fraction was normalized as variation from its lowest value (see Figure S15).

### Statistical Analysis

All statistical assays performed were analyzed using analysis of variance and then Tukey’s test using OriginJ software v1.52i (NIH, USA). *P*-values <0.05 were considered statistically significant.

## Supporting Information Available

Additional experimental details, including materials and methods, details of data and image analysis, and supporting results including cell viability, supporting cryo-TEM images and relative analysis, additional confocal microscopy of GUVs and air exposed biofilm, supplementary calcein release assay, long-term stability of liposomal dispersions, and tables summarizing the stability of liposomes at different conditions (DLS) (PDF)
