# Supplementary material for: Designer Liposomic Nanocarriers Are Effective Biofilm Eradicators
Source: ACS Nano. 2022 Aug 26;16(10):15792–804. doi: 10.1021/acsnano.2c04232 (PMC9620068; doi:10.1021/acsnano.2c04232)
Supplement: Supplementary file 1 — nn2c04232_si_001.pdf [file nn2c04232_si_001.pdf]

Supplementary Materials for

Designer liposomic nano-carriers are effective biofilm eradicators

Monika Kluzek[1‡], Yaara Oppenheimer-Shaanan[1‡], Tali Dadosh[2], Mattia I. Morandi[3], Ori Avinoam[3], Calanit Raanan[4], Moshe Goldsmith[3], Ronit Goldberg[1†], Jacob Klein[1*]*

[1]Department of Materials and Interfaces, Weizmann Institute of Science, Rehovot, 76100, Israel

[2]Department of Chemical Research Support, Weizmann Institute of Science, Rehovot, 76100, Israel

[3]Department of Biomolecular Sciences, Weizmann Institute of Science, Rehovot, 76100, Israel

[4]Department of Veterinary Resources, Weizmann Institute of Science, Rehovot, 76100, Israel

* monika.kluzek@weizmann.ac.il; jacob.klein@weizmann.ac.il

This PDF file includes:

Materials and methods

Figures S1 to S15

Tables S1 to S3

References (1-3)

Table of content

Materials and methods P. 4

Fig. S1: Changes in hydrodynamic diameter of HSPC (50%) / cholesterol (40%) / stearylamine (5%) / pMPC (5 kDa, 5%) liposomes upon storage at 4 ˚C. P. 6

Fig. S2: Cell viability of HEK293 cells upon 24 h-incubation with antimicrobial agents (SMX and SMX/EA) free and encapsulated in pMPC- and PEG-functionalized liposomes. P. 7

Fig. S3: Histograms of distribution of distance between opposing vesicles measured based on cryo-TEM pictures. P. 8

Fig. S4: Confocal microscopy images of time-lapse acquisition of pMPC- giant unilamellar vesicles (GUVs) stained with DiI dye (red), interacting in HEPES-glucose buffer. P. 9

Fig. S5: Picture of non-functionalized liposomes composed of HSPC (60%) / cholesterol (40%) (left Eppendorf) and same composition doped with 5% stearylamine (right Eppendorf). P. 10

Fig. S6: Changes in hydrodynamic diameter of non-functionalized liposomes composed of (A) HSPC (60%) / cholesterol (40%) and (B) HSPC (55%) / cholesterol (40%) / stearylamine (5%) upon storage at 4 ˚C. P. 11

Fig. S7: Effect of liposome diameter and stearylamine presence on calcein release profiles upon interaction between liposomes and PA14 at 37˚C. P. 12

Fig. S8: Picture of pMPC-liposomes loaded with SMX/EA. P. 13

Fig. S9: Representative cryo-TEM images of (A) liposomes loaded with SMX and (B-C), co-loaded with SMX/EA. Scale bar, 50 nm. P. 14

Fig. S10: Release profiles of SMX and SMX/EA loaded into PEG-functionalized liposomes at physiological condition (37 ˚C, pH 7.2). Results are shown as average and standard deviation of 3 independent experiments. Dashed lines represent trend of the data points. P. 15

Fig. S11: Retention profiles of loaded drugs inside pMPC-liposomes upon storage at 4˚C. P. 16

Fig. S12: Correlation between resazurin assay (I585/ I635) and CFU estimated on growth of PA14 strain. P. 17

Fig. S13: CFU/mL of PA14-biofilm after two-dose treatment with SMX- and SMX/EA loaded liposomes and free drugs. P. 18

Fig. S14: Inhibition effect of antimicrobial agents (SMX, SMX/EA) encapsulated in functionalized liposomes on pyocyanin (a virulence factor with several physiological roles in *P. aeruginosa* biofilms) production in PA14-biofilm. P. 19

Fig. S15: Schematics showing the data analysis workflow for quantification of LIVE (green) / DEAD (red) signal in images of paraffin-embedded and 10 µm-thin section of colony upon treatment with 5 µL PEG-LUVs loaded with SMX. P. 20

Table S1: Zeta potential and hydrodynamic diameter of pMPC-LUVs and PEG-LUVs drug-free and loaded with either sulfamethoxazole (SMX, drug) or co-loaded with sulfamethoxazole and ellagic acid (SMX/EA). P. 21

Table S2: Effect of BM2G medium on hydrodynamic diameter of pMPC-LUVs and PEG-LUVs. P. 22

Table S3: Chemical and physical properties of sulfamethoxazole (SMX) and ellagic acid (EA). P. 23

References P. 24

Materials and methods

Evaluation of cytotoxicity

Liposomes cytotoxicity was determined by the production of the yellow formazan product upon cleavage of XTT by mitochondrial dehydrogenases in viable HEK 293 cells (human embryonic kidney). The cells were seeded onto 96-well plates (4×10^4 cells/well) in DMEM media. When the confluent state was reached (usually after 24 h), 50 μL of pMPC-, PEG- liposomes and SMX (1.5 mg/mL) or SMX (0.7 mg/mL)/EA(1.5 mg/mL) mixture in PBS solutions were then added. After 24 h incubation, the cells were incubated with 50 μL of XTT solution composed for 3 h. Absorbance values were later measured with a multiwell-plate reader (Cary 100 Bio, Varian Inc, USA) at a wavelength of 450 nm. Background absorbance was measured at 620 nm and subtracted from the 450 nm measurement. The experiments were repeated at least three times, and six replicates were prepared for each liposomes concentration tested in every experiment. A solution in DMEM culture medium was used as a positive control.

The potential toxic effect of the different liposomal formulations tested was expressed as a viability percentage calculated using the following formula:

$$\%Viability=100-[(ODtest/ODc)\times100]$$

Where ODtest was the optical density of those wells treated with the liposomes solutions, and ODc was the optical density of those wells treated with supplement-free DMEM media.

Pyocyanin quantitation assay

Pseudomonas aeruginosa (PA) biofilms were grown on 24-well plate. 1 mL of the inoculum was grown in a humidified incubator for 24 h at 37 ˚C without shaking. After 24h, the fully formed biofilm was gently washed two times with PBS. 1 mL of liposomal suspension or BM2G medium was added to each well and subsequently the samples were incubated at 37 ˚C for 4 h. Each well was then washed with PBS and second does (1 mL) of antibacterial treatment was applied for 4 h with incubation at 37 ˚C. Subsequently 24-well plate was sonicated at room

temperature for 5 min. The collected bacteria suspension were centrifuged at 4500 rpm for 20 min. Obtained supernate was filtrated through 0.22 μm syringe filter. 0.5 mL of supernate was mixed with 0.17 mL of chloroform and shaken vigorously. Subsequently, samples were centrifuged at 4500 rmp for 10 min. The organic phase was transfer to new tubes and mixed with 0.12 mL of 0.2 M HCl and shaken vigoursly. The Samples were again centrifuged at 4500 rpm after which water phase was collected and pyocyanin was quantified using HPLC analysis.

Pyocyanin analysis by HPLC

Samples and pyocyanin standard solutions were prepared in 0.2 M HCl, supplemented with 1,5-Naphthalenediamine (0.1 mg/mL) as internal standard and filtered through 0.2 mm PTFE filters (StarTech®, CA) prior to HPLC analysis. Sample solutions were kept at 4˚C prior to injection and were separated by reverse-phase chromatography on a Gemini C18 (4.6 x 150 mm, 5 mm) column (Phenomenex©, USA). Chromatography was performed on a Prominence UFLC LC-20AD system (Shimadzu©, Japan) consisting of a SIL-20AC autosampler (Shimadzu©, Japan), CTO-20AC column oven (Shimadzu©, Japan) and a SPD-M20A diode array detector (Shimadzu©, Japan). Elution was done using an isocratic gradient of 70% solvent A (0.04% TFA in water) and 30% solvent B (10% water and 0.04% TFA in acetonitrile) at a flow rate of 1 mL per minute for 5 minutes at 25 ˚C, while monitoring at 388nm. Data analysis was performed using LabSolutions ver. 5.97 (Shimadzu©, Japan).

Stability of loaded liposomes

Liposomes with encapsulated drug were store at 4 ˚C, and at define time points, liposomal suspension was dialyzed (dialysis bag with 50 kDa cutoff) against Na_2SO_4 (pH 7.0, ~320 mOsmo/kg). Amount of remining drug encapsulated in liposomes was determined using ultraviolet spectrophotometer (Cary 100 Bio, Varian Inc, USA).

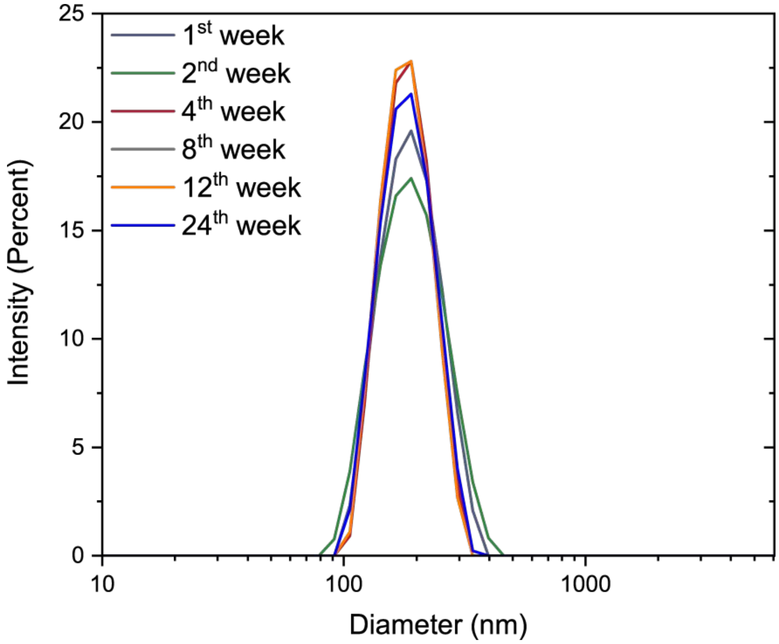

Fig. S1.

Changes in hydrodynamic diameter of HSPC (50%) / cholesterol (40%) / stearylamine (5%) / pMPC (5 kDa, 5%) liposomes (extruded through 200 nm pore membrane) upon storage at 4 ˚C.

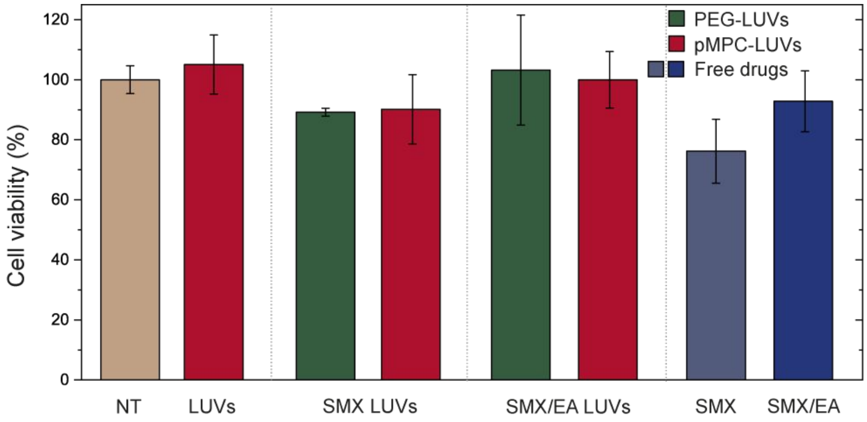

Fig. S2.

Cell viability of HEK293 cells upon 24 h-incubation with antimicrobial agents (SMX and SMX/EA) free and encapsulated in pMPC- and PEG-functionalized liposomes. Liposomes were extruded through 200 nm pore membrane. Cell viability was measured with a Cell Proliferation Kit (XTT based) following the manufacturer instruction. The data represents the averages and standard deviations from two independent biological repeats. Blue bars (SMX, SMX/EA) treatment with free drugs. Abbreviation: NT-not treated.

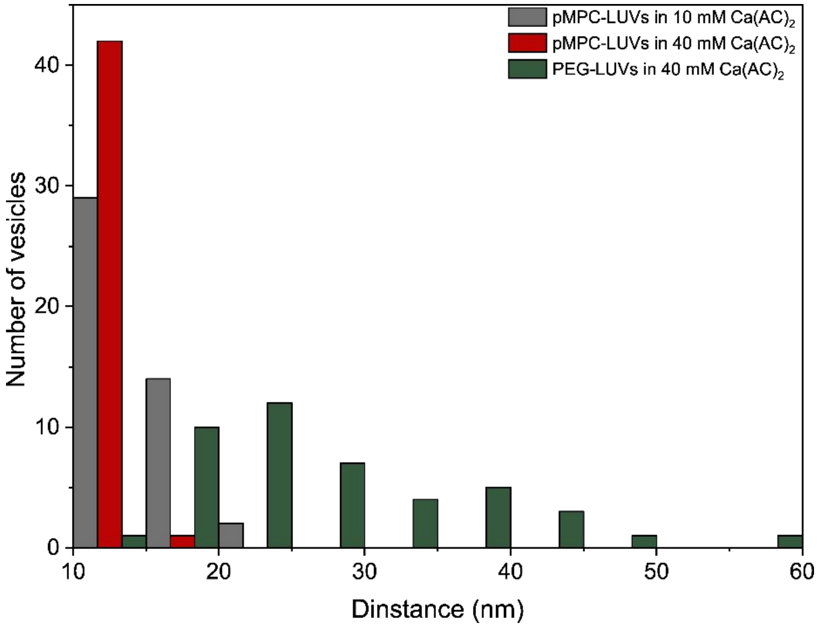

Fig. S3.

Histograms of distribution of distance between opposing vesicles measured based on cryo-TEM pictures. The distance between membranes was measured considering the length between the phospholipid headgroups of the inner leaflet of the two vesicles. The data represent measurements extracted from 7 separate fields of view, for a total of N = 45 for pMPC-LUVs in 10 mM $Ca(Ac)_2$ pMPC; N=43 for pMPC-LUVs in 40 mM $Ca(Ac)_2$ and N=44 for PEG-LUVs in 40 mM $Ca(Ac)_2$.

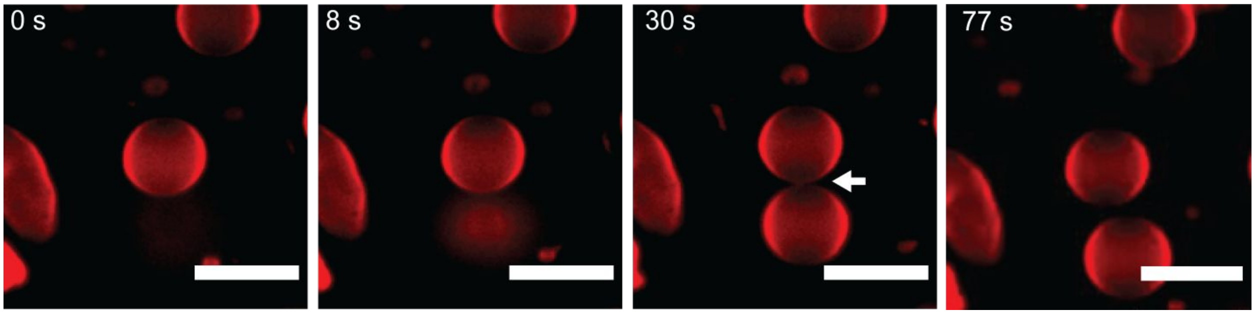

Fig. S4.

Confocal microscopy images of time-lapse acquisition of pMPC- giant unilamellar vesicles (GUVs) stained with DiI dye (red), interacting in HEPES-glucose buffer. Scale bar, 20 μm. White arrow indicate interaction points.

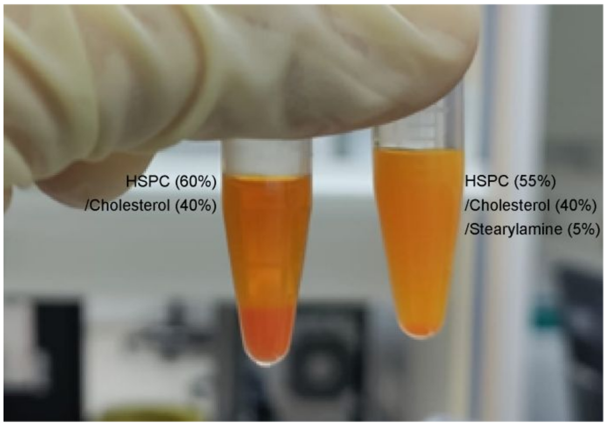

Fig. S5.

Picture of non-functionalized liposomes composed of HSPC (60%) / cholesterol (40%) (left Eppendorf) and same composition doped with 5% stearylamine (right Eppendorf). Both samples were calcein-loaded and images were taken 3 days post-extrusion upon storage at 4˚C.

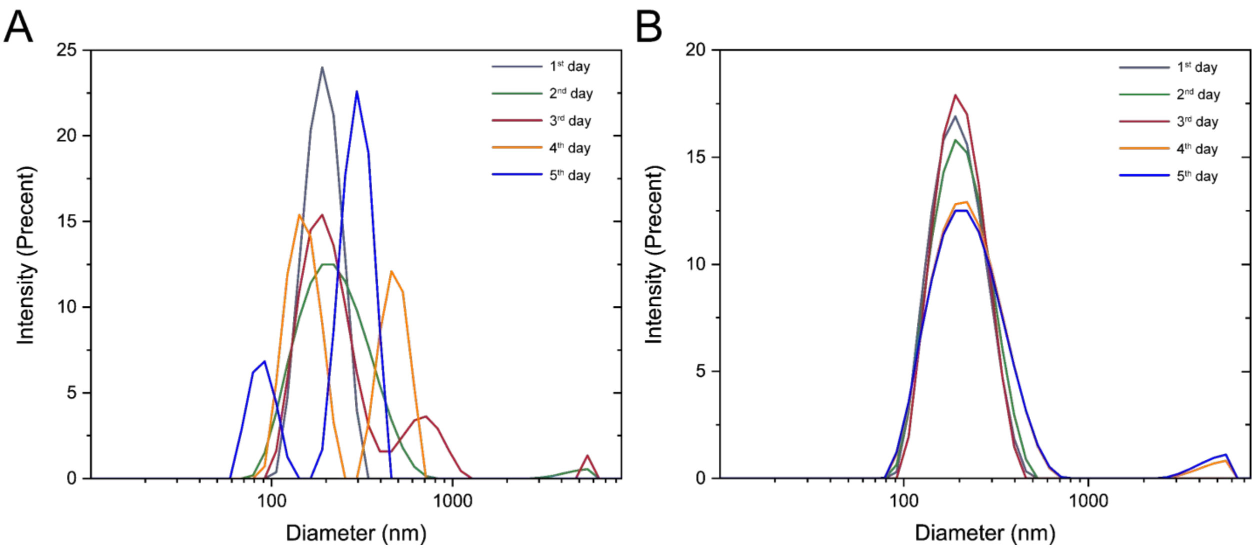

Fig. S6.

Changes in hydrodynamic diameter of non-functionalized liposomes composed of (A) HSPC (60%) / cholesterol (40%) and (B) HSPC (55%) / cholesterol (40%) / stearylamine (5%) (extruded through 200 nm pore membrane) upon storage at 4 ˚C.

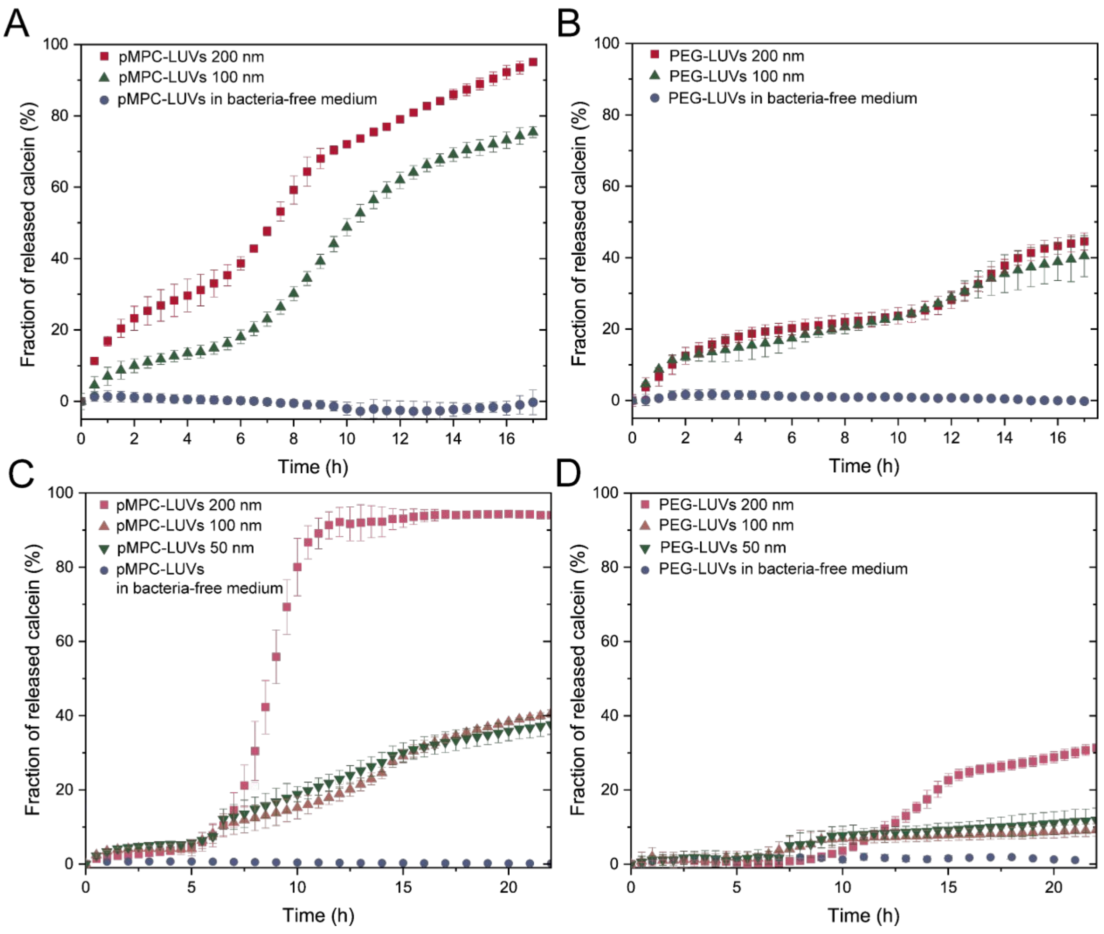

Fig. S7.

Effect of liposome diameter and stearylamine presence on calcein release profiles upon interaction between liposomes and PA14 at 37˚C. Liposomes were functionalized with pMPC- (**A,C**) or PEG- (**B, D**) polymer (5%) and extruded through 200 nm, 100 nm or 50 nm pore membrane. Release of calcein in biofilm was compared between LUVs with no positive charge in the membranes (**C, D**) and containing 5% mol of cationic lipid stearylamine (SA) (**A, B**), demonstrating that a rapid cargo release requires the presence of positively charged lipids (SA),[1–3] as incubation with LUVs lacking SA still shows calcein release, but with a characteristic lag time of 400 min (Fig. S7 C, D). LUVs extruded through 200 nm pore membrane encapsulating calcein were incubated with BM2G media only as a negative control (LUVs in bacteria-free medium). Results are from a minimum of 3 independent experiments.

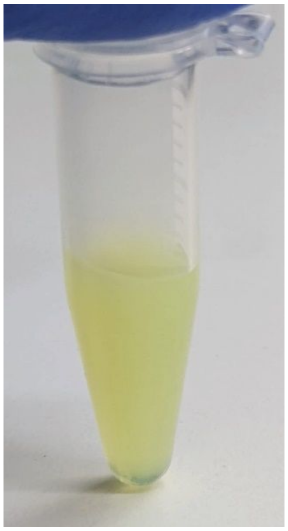

Fig. S8.

Picture of pMPC-liposomes loaded with SMX/EA.

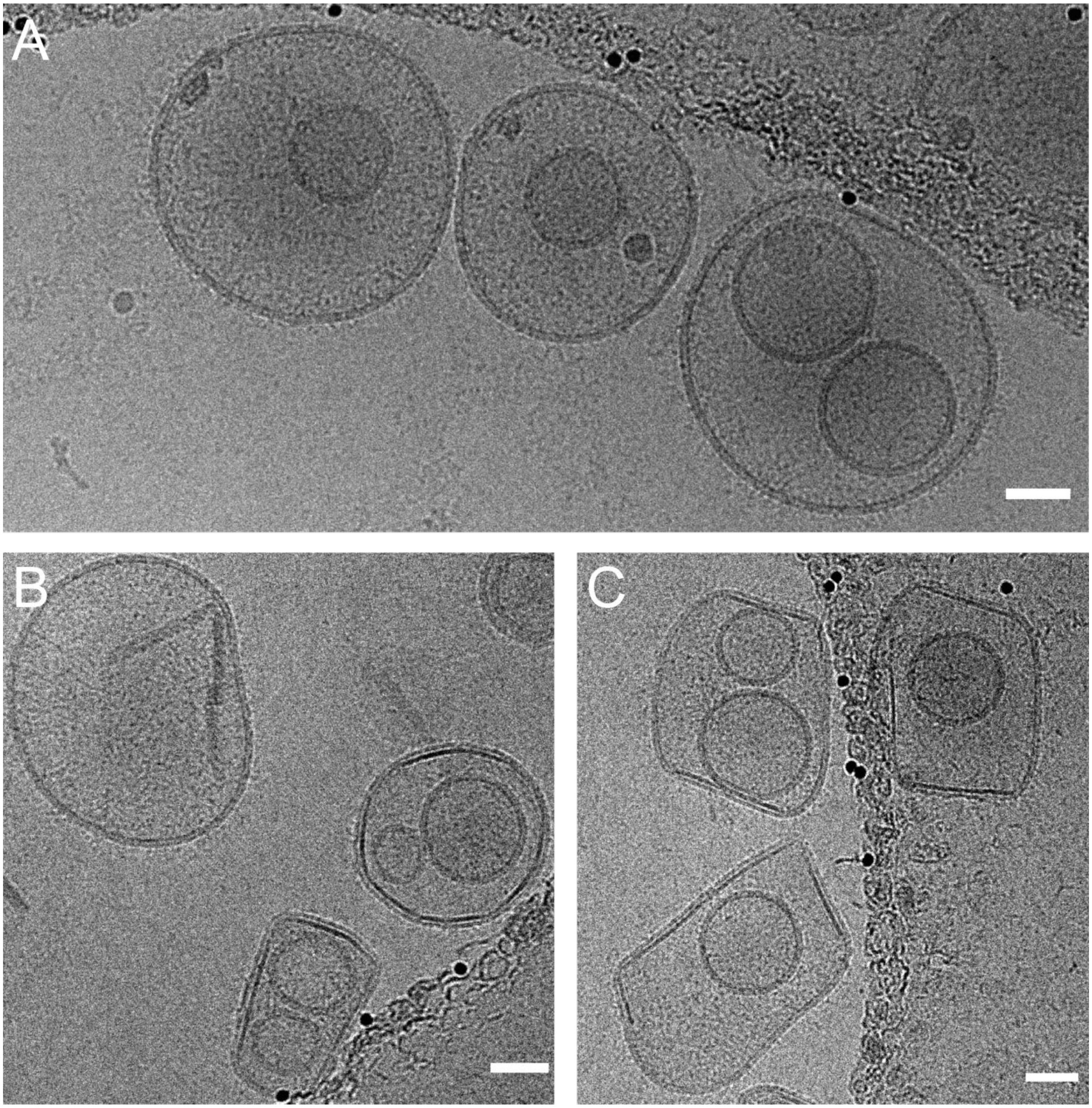

Fig. S9.

Representative cryo-TEM images of (**A**) liposomes loaded with SMX and (**B**-**C**), co-loaded with SMX/EA. Scale bar, 50 nm.

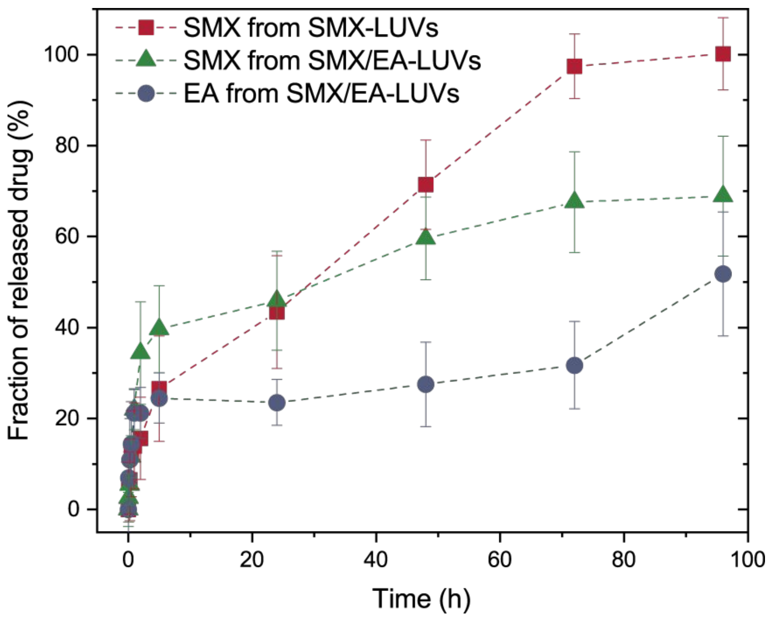

Fig. S10.

Release profiles of SMX and SMX/EA loaded into PEG-functionalized liposomes at physiological condition (37 ˚C, pH 7.2). Results are shown as average and standard deviation of 3 independent experiments. Dashed lines represent trend of the data points.

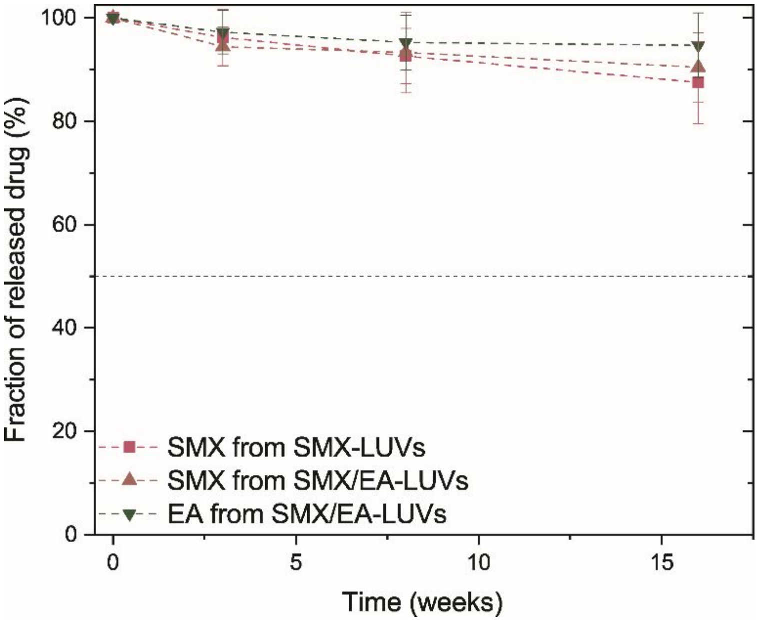

Fig. S11.

Retention profiles of loaded drugs inside pMPC-liposomes upon storage at 4˚C. Results are from a minimum of 2 independent experiments with 3 technical repeats.

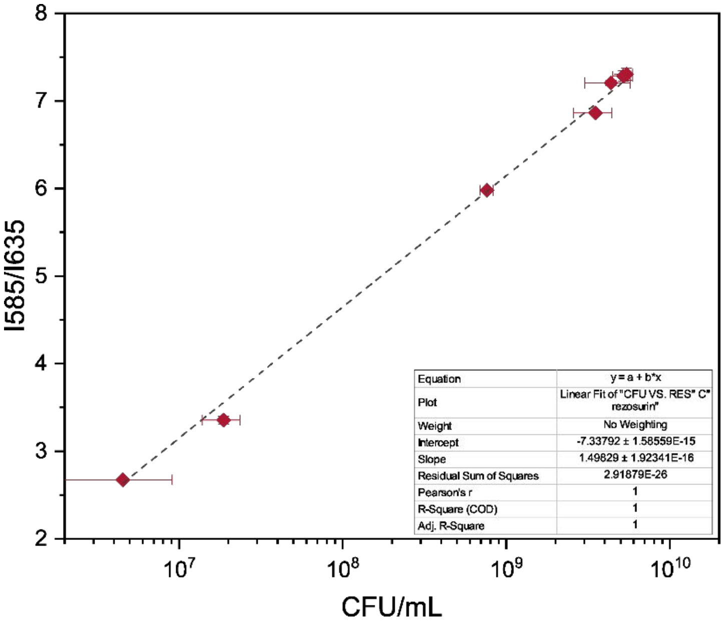

Fig. S12.

Correlation between resazurin assay (I585/ I635) and CFU estimated on growth of PA14 strain.

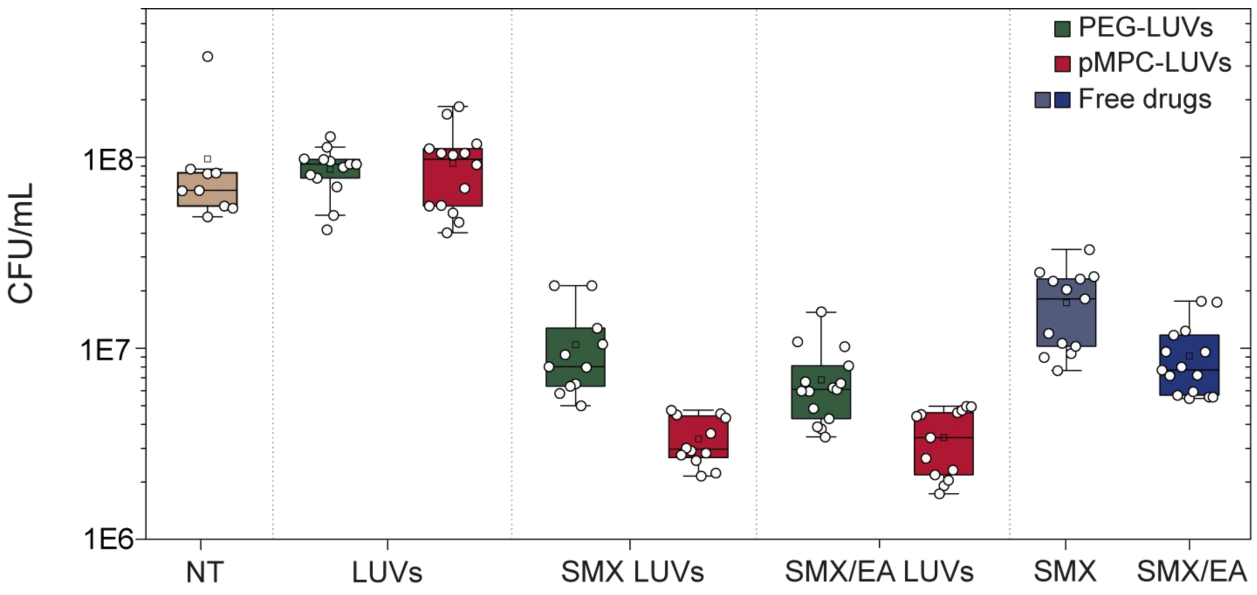

Fig. S13.

CFU/mL of PA14-biofilm after two-dose treatment with SMX- and SMX/EA loaded liposomes and free drugs. CFU results are calculated from correlation between resazurin assay and CFU from Fig S10. Biofilm was grown on a MBEC^{TM} assay lid, and after two doses of antimicrobial treatment the remaining cells were removed to fresh BM2G by sonication and cell viability was quantified by resazurin assay after 400 min incubation. Blue boxes (SMX, SMX/EA) represent treatment with free drugs. Abbreviation: NT-not treated biofilm. Differences between groups shown in the box plot were tested with a one-way ANOVA. Boxes represent the 25-75 percentiles of the sample distribution, with black vertical lines representing the 1.5×IQR (interquartile range). Black horizontal line represents the median.

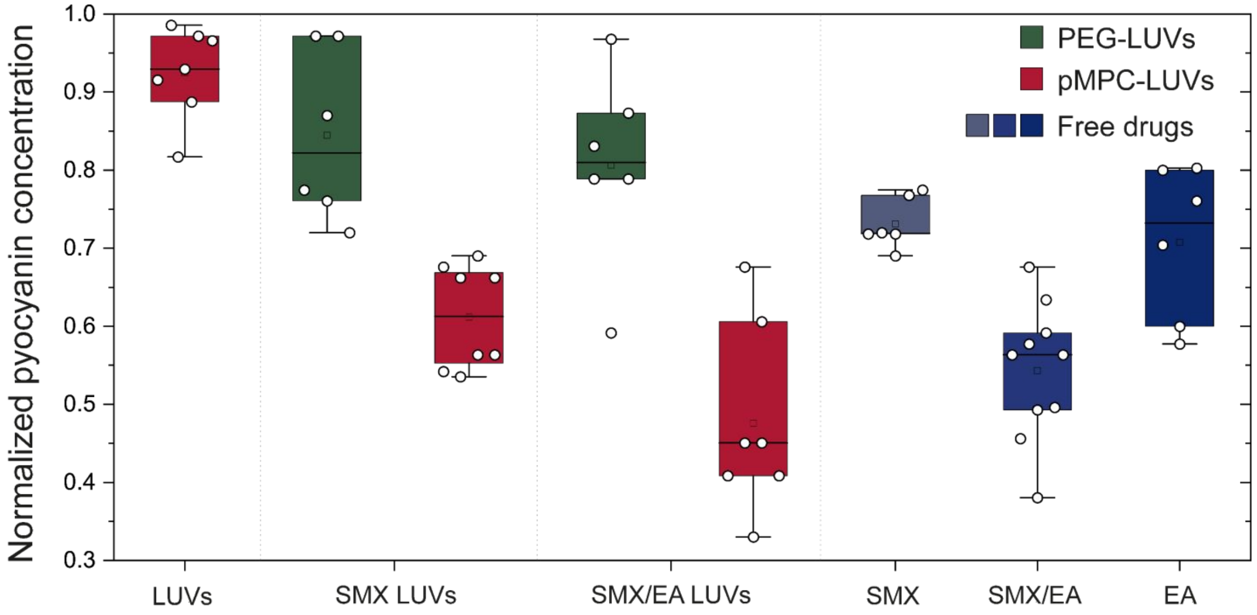

Fig. S14.

Inhibition effect of antimicrobial agents (SMX, SMX/EA) encapsulated in functionalized liposomes on pyocyanin (a virulence factor with several physiological roles in *P. aeruginosa* biofilms) production in PA14-biofilm. Results clearly show that delivery of EA induces a significant reduction in pyocyanin production, with 20% decrease already observed for administered free EA-only. Normalized pyocyanin concentration measured by HPLC method

after 4 h incubation with one dose of a treatment. The data represents minimum 3 biological repeats. Boxes represent the 25-75 percentiles of the sample distribution, with black vertical lines representing the 1.5×IQR (interquartile range). Black horizontal line represents the median.

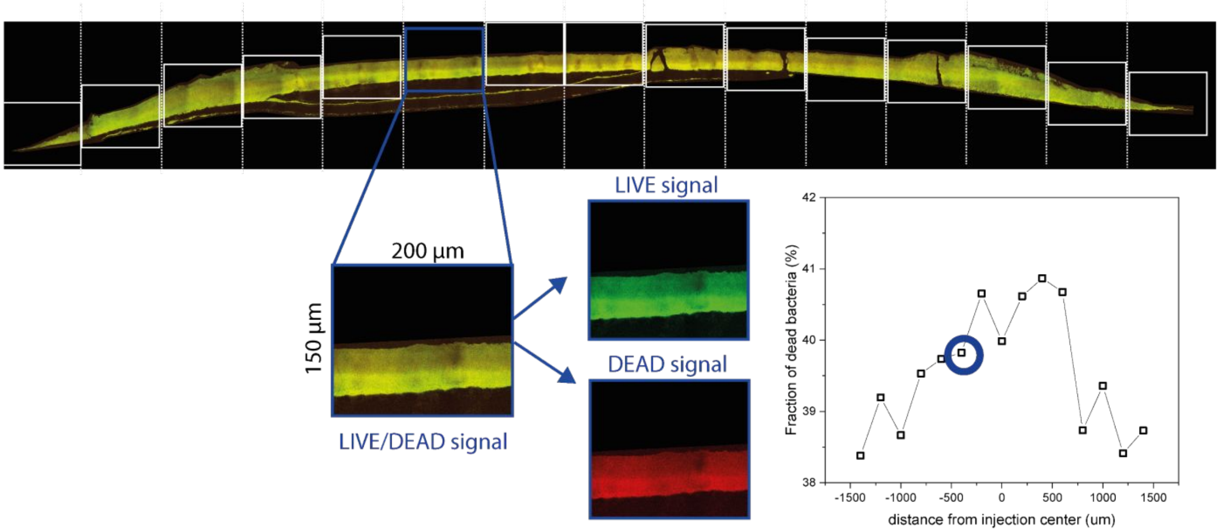

Fig. S15.

Schematics showing the data analysis workflow for quantification of LIVE (green) / DEAD (red) signal in images of paraffin-embedded and 10 µm-thin section of colony upon treatment with 5 µL PEG-LUVs loaded with SMX. Images were contoured to isolate only the biofilm section and remove the fluorescent background signal. Resulting composite images were segmented with a 200 µm wide and 150-200 µm high region of interest. For each segment, average intensity of the two channels (green / red) was measured and the fraction of dead bacteria was quantified as intensity red channel over the sum of both channels. To compare different biofilm sections, each profile of dead bacteria fraction was normalized as variation from its lowest value.

Sample	Unloaded			SMX		SMX/EA	
	Diameter (nm)	PDI	ζ-potential (mV)	Diameter (nm)	PDI	Diameter (nm)	PDI
HSPC (50%) / cholesterol (40%) / stearylamine (5%) / pMPC (5 kDa, 5%)	179.7	0.04	-4.24 ± 0.02	181.5	0.05	189.4	0.19
HSPC (50%) / cholesterol (40%) / stearylamine (5%) / PEG (5 kDa, 5%)	165.5	0.04	-1.84 ± 0.08	164.9	0.05	171.4	0.12

Table S1.

Zeta potential and hydrodynamic diameter of pMPC-LUVs and PEG-LUVs (extruded through 200 nm membrane) drug-free and loaded with either sulfamethoxazole (SMX, drug) or co-loaded with sulfamethoxazole and ellagic acid (SMX/EA). Values are shown as averages over 3 samples with 10 runs each.

Sample	Na_2SO_4		BM2G	
	Diameter (nm)	PDI	Diameter (nm)	PDI
HSPC (50%) / cholesterol (40%) / stearylamine (5%) / pMPC (5kDa, 5%)	189.8	0.08	185.0	0.10
HSPC (50%) / cholesterol (40%) / stearylamine (5%) / PEG (5kDa, 5%)	161.4	0.08	159.3	0.08

Table S2.

Effect of BM2G medium on hydrodynamic diameter of pMPC-LUVs and PEG-LUVs, extruded through 200 nm pore membrane. Values are shown as averages over 3 samples with 10 runs each, and the standard deviation is used as an error estimate.

	Mw [g/mol]	Water solubility [mg/mL]	logP	pK_a
Sulfamethoxazole (SMX)	253.3	0.46 – 0.61	0.7- 0.89	5.7
Ellagic acid (EA)	302.2	0.82	1.59- 2.32	5.8

Table S3.

Chemical and physical properties of sulfamethoxazole (SMX) and ellagic acid (EA) adapted from PubChem [www.pubchem.ncbi.nlm.nih.gov].

References

(1) Drulis-Kawa, Z.; Dorotkiewicz-Jach, A.; Gubernator, J.; Gula, G.; Bocer, T.; Doroszkiewicz, W. The Interaction between Pseudomonas Aeruginosa Cells and Cationic PC:Chol:DOTAP Liposomal Vesicles versus Outer-Membrane Structure and Envelope Properties of Bacterial Cell. *Int J Pharmaceut* 2009, *367* (1–2), 211–219.

(2) Nicolosi, D.; Scalia, M.; Nicolosi, V. M.; Pignatello, R. Encapsulation in Fusogenic Liposomes Broadens the Spectrum of Action of Vancomycin against Gram-Negative Bacteria. *Int J Antimicrob Ag* 2010, *35* (6), 553–558.

(3) Ma, Y.; Wang, Z.; Zhao; Lu, T.; Wang, R.; Mei, Q.; Chen, T. Enhanced Bactericidal Potency of Nanoliposomes by Modification of the Fusion Activity between Liposomes and Bacterium. *Int J Nanomed* 2013, *Volume 8* (1), 2351–2360.
